# Soil Microbial Assembly Mirrors a Savanna Mosaic Amid Plant Invasion and Soil Disturbance

**DOI:** 10.1002/ece3.74284

**Published:** 2026-09-03

**Authors:** Elizabeth A. Bowman, A. Peyton Smith, Aaron C. Rhodes, Robert M. Plowes, Lawrence E. Gilbert

**Affiliations:** ^1^ Brackenridge Field Laboratory University of Texas at Austin Austin Texas USA; ^2^ Department of Soil and Crop Sciences Texas A&M College Station Texas USA; ^3^ Department of Integrative Biology University of Texas at Austin Austin Texas USA

**Keywords:** bacteria, community assembly, fungi, *Megathyrsus maximus*, mesquite, plant–soil interactions, savanna, soil microbial community

## Abstract

Savannas, spanning 20% of the Earth's surface, are characterized by a continuous grass matrix interspersed with woody patches, supporting high biodiversity and providing ecological and economic services. Although climatic and edaphic controls on savanna structure are well studied, the contribution of soil microbial communities in maintaining spatial heterogeneity and coexistence remains poorly understood. We investigated whether savanna heterogeneity is mirrored belowground and how disturbance and invasion by *Megathyrsus maximus* can modify these relationships. We used a factorial field sampling design in a mesquite savanna to compare woody patches and adjacent grasslands with and without mechanical disturbance and invasion. We quantified soil physicochemical properties, plant community composition, and bacterial and fungal communities to evaluate linkages among vegetation and soils. Grasslands and woody patches supported distinct soil and microbial assemblages, consistent with differences in vegetation inputs and nutrient regimes. Grassland microbial communities exhibited relatively simple assembly patterns, with plant diversity and soil chemistry serving as the primary correlates of community composition and stronger associations observed for fungi than bacteria. In woody patches, soil physicochemical properties were the primary correlates of microbial community composition, with plant diversity exhibiting weaker associations than in grasslands. In grasslands, invasion by *M. maximus* was associated with higher soil nutrient availability and shifts in microbial community composition, and mechanical disturbance correlated with similar but weaker effects. These shifts aligned with reduced grassland microbial distinctiveness and may disrupt linkages between vegetation, soils, and microbes. Aboveground patch structure in savannas aligned with belowground microbial communities and nutrient dynamics. By demonstrating that invasion and disturbance weaken these spatial patterns, this study indicates that soil microbial communities may contribute to savanna coexistence and resilience, revealing a possible association between belowground community assembly and landscape‐scale heterogeneity and its destabilization under multiple stressors.

## Graphical abstract

Grasslands and woody patches supported distinct soil and microbial assemblages, consistent with differences in vegetation inputs and nutrient regimes. Grasslands exhibited relatively simple assembly patterns, with plant diversity closely associated with soil nutrients and comparatively homogeneous microbial communities. In contrast, woody patches displayed more complex assembly dynamics, with microbial communities structured by plant diversity, soil nutrients, and spatial distance, indicating greater environmental heterogeneity and stronger dispersal limitation.

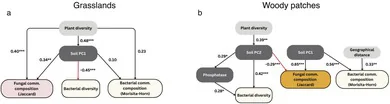

## 1 Introduction

Savannas span approximately 20% of the Earth's land surface, covering substantial portions of the surface area in Africa, Australia, and South America (Grace et al. 2006). Responsible for approximately 30% of global net primary productivity and providing pastureland for a majority of the world's livestock, savannas serve important ecological and economic functions (Mishra and Young 2020). Characterized by a continuous grassland matrix with patches of woody plants, savannas are unique ecosystems that host high levels of biodiversity (Mishra and Young 2020). However, the codominance of woody and herbaceous plant communities can make these ecosystems unstable and at risk of loss with increasing manipulation by humans, biological invasion, and climate‐change related processes (Biancari et al. 2024).

Savannas vary both geographically and temporally in the ratio of woody to herbaceous species, but the coexistence of these two plant communities within close proximity is thought to be maintained by stabilizing interactions of climate (particularly precipitation), soil nutrients, and disturbance (Bond 2008; Frost et al. 1986; Sankaran et al. 2004, 2005; Scholes and Archer 1997; Van Langevelde et al. 2010). Water availability can play an important role in the stabilization or destabilization of savanna systems; for instance, a mean annual precipitation of approximately 650 mm limits woody plant establishment and growth in African savannas, allowing for the coexistence of grasses (Hanan and Lehmann 2010; Sankaran et al. 2005). Disturbance, particularly fire and herbivory, acts as top‐down regulators of woody plant cover, limiting woody plant establishment, although interactions are complex (Bond 2008; Scholes and Archer 1997). In general, savannas are categorized as having highly weathered, nutrient‐poor soils (Bond 2008), but the woody patches provide an important influx of nutrients (Scholes and Archer 1997). The relative contribution of climate, soil nutrients, or disturbance in structuring savannas likely varies over both time and space. Despite a wide literature on savannas, little attention has been paid to belowground microbial communities and their potential contributions to the stability dynamics of these systems.

Phylogenetically and functionally diverse, soil microbial communities are important drivers of nutrient cycling and plant community succession (Wardle et al. 2004; Van Der Heijden et al. 2008; Bever et al. 2010). At a regional and landscape scale, climate, edaphic patterns, and land use history drive the selection and filtering of microbial communities (de Vries et al. 2012; Drenovsky et al. 2010; Tedersoo et al. 2014; Větrovský et al. 2019). Selection of the microbial community at a local scale is driven by finer‐scale variation in soil nutrients, plant litter, and other exudates released into the soil by distinct plant species and lineages impacting communities of decomposers, mutualists, and pathogens (Hiiesalu et al. 2014; Leff et al. 2018). The grassland matrix of savannas is composed of plant species that are phylogenetically and functionally distinct from species found in woody patches; as a result, these two vegetation types likely host distinct microbial communities (de Vries et al. 2012). For instance, grasses and herbaceous species of plants associate predominantly with arbuscular mycorrhizal fungi (subphylum: Glomeromycotina) and dark‐septate endophytes on their roots (phylum: Ascomycota) (S. Smith and Read 2008). In contrast, the dominant trees in many savannas are members of the plant family Fabacaeae which associates with nitrogen‐fixing bacteria on their roots (e.g., *Vachellia* and *Senegalia* spp., *Lonchocarpus nelsii*, *Brachystegia* spp. in African savannas; *Mimosa* spp. in Cerrados of South America; *Vachellia* spp. and *Mimosa* spp. in India; *Acacia* spp. and *Bauhinia* spp. in Australia; *Prosopis* and *Vachellia* spp. in North America) (Mishra and Young 2020; Simon et al. 2009). Nitrogen‐fixing woody species create fertile islands within savannas with availability of soil nutrients increasing with the establishment and expansion of woody plants (Scholes and Archer 1997). An experiment of responses of grassland microbial communities across the globe to increases in nitrogen and phosphorus showed that soil communities tend to lose plant mutualists and increase in the abundance of free‐living microbial communities (Leff et al. 2015). Structural changes in the plant community of savannas are likely to correlate with shifts in the soil microbial community. These changes may be exacerbated by invasive species, which can alter microbial communities and associated processes such as plant–soil feedback.

Invasive plants alter ecosystem function and aboveground biodiversity in a variety of ecosystems (Hickman et al. 2013; Pyšek et al. 2012; Torres et al. 2021), but little is known about how invasion alters microbial communities in savannas. *Megathyrsus maximus* (Jacq.) B.K. Simon and Jacobs, Guinea grass, is a pantropically distributed invasive grass that alters plant community composition and produces abundant biomass, reducing native plant diversity, and increasing litter deposition (Rhodes et al. 2025; Simon and Jacobs 2019). *M. maximus* exudes deleterious allelochemicals into soils, which reduce native plant germination and establishment (Morrison et al. 2023). Initially, *M. maximus* invades highly disturbed grasslands and nutrient‐rich woody patches before introgression into native grasslands (Rhodes et al. 2022). In invaded grasslands, *M.maximus* invasion was reinforced with positive plant–soil feedback processes, whereas negative plant–soil feedback in native grasslands helped maintain species coexistence and high levels of diversity (Bowman et al. 2023). Each of these traits of *M. maximus* may act to destabilize soil microbial communities and alter successional pathways.

Despite a high level of microbial diversity in soils, local adaptation of plants, soil, and microbial symbionts with sympatric relationships occurs rapidly (Johnson et al. 2010; Revillini et al. 2016; Rúa et al. 2016). Plants filter and recruit specific microbes in their own rhizosphere such as arbuscular mycorrhizal fungi or nitrogen‐fixing bacteria for members of the family Fabaceae, which affect the host plant's access to nutrients and growth (Revillini et al. 2016). In bulk soil, changes in the community of decomposers can arise from alterations in litter quality and root exudates during a plant invasion impacting nutrient cycling (Wolfe and Klironomos 2005). Because these microbial shifts can influence nutrient availability, vegetation dynamics, and ecosystem feedbacks, soil microbial communities may play a substantial role in accelerating or slowing successional trajectories within savannas.

In this study, our objective was to describe variations in abiotic and biotic soil properties of a mesquite savanna in response to several potential drivers. We assessed changes in soil nutrient and physical characteristics in tandem with fungal and bacterial communities across mesquite‐dominated woody patches and grasslands, assessing how they were affected by mechanical disturbance and invasion by *M. maximus* . Furthermore, we aimed to evaluate to what extent disturbance, *M. maximus* invasion, and savanna structure are correlated to differences in soil biotic and abiotic characteristics. Specifically, we asked three questions:

1. how do soil properties and microbial communities vary across the woody patches and grassland matrix that compose biphasic mesquite savannas?
2. how are these patterns disrupted by mechanical disturbance and invasion by *M. maximus* (i.e., are there similar responses in both grasslands and woody patches)?
3. to what extent do bacterial and fungal communities shift in parallel with each other and soil nutrients across savanna structure?

To answer these questions, we conducted an observational field study using a full factorial sampling design to assess biotic and abiotic soil properties associated with the three focal factors (mechanical disturbance, invasion, and savanna structure). Ultimately, our goal was to determine how belowground processes can inform our understanding of community dynamics in savannas and be incorporated into models of succession. By examining fungal and bacterial communities alongside plant communities and soil characteristics, this study examines relationships among above‐ and belowground components in a savanna ecosystem during a plant invasion.

## 2 Materials and Methods

Spanning desert, coastal, and subtropical ecoregions, South Texas rangelands are home to diverse communities of plants and animals (Wied et al. 2020). Much of the ecoregion is composed of mesquite savannas that form heterogeneous landscapes consisting of a biphasic grassland matrix with woody patches dominated by N‐fixing *Prosopis* spp., which we refer to as the two vegetation types in this study. These savannas may undergo natural succession over long‐term time scales (400–500 years) from grasslands to woody plant‐dominated communities during low disturbance regimes, with possible intermediate stages of dense immature mesquite shrubs if past natural state changes followed the scenario seen after the disturbance of anthropogenic woody plant removal (Archer 1995; Whittaker et al. 1979). Overgrazing and suppression of fire regimes have increased the rate of expansion by woody plant species (Archer 1995), and in order to maintain grasslands for grazing, mechanical removal of woody species (i.e., mechanical disturbance) is used to slow woody introgression but may have limited long‐term success. Facilitated by this disturbance, invasive non‐natives like *M. maximus* are becoming more prevalent within these grasslands, leading to a decrease in habitat for native plant and animal species (Rhodes et al. 2021).

We sampled across three sites in Kenedy and Brooks County, Texas which spanned 33 km^2^ with the distance between sites ranging from 6 to 10 km on a ranch with documented fire, grazing, and brush management practices. All sites were selected to be in the same soil series, the Palobia loamy fine sand described as a fine‐loamy, mixed, active, hyperthemic Typic Natrustalf (Soil Survey Staff et al. 2024). Within each site, we collected soil samples and plant assemblage data from 100 m^2^ plots for each cross (2 disturbance types × 2 invasion types × 2 vegetation types; Appendix S2: Table S1) with treatments being vegetation (grassland vs. woody), invasion status by *M. maximus* (invaded vs. non‐invaded), and disturbance (time since mechanical brush clearing, recent vs. pre‐1980), as well as their interactions. Plots were selected during an initial visual survey to be homogenous across sites for these treatment factors. Our sampling scheme is further described in Appendix S1. Mean annual precipitation (MAP) and mean annual temperature (MAT) were initially extracted at a 30 s spatial resolution (approx. 1 km^2^) from WorldClim version 2.1 (Fick and Hijmans 2017). However, because the spatial resolution of these data was substantially coarser than our 33 km^2^ study area, climatic variables were excluded from the final analyzes to avoid potential scale mismatch among plots (Appendix S2: Figure S1).

### 2.1 Plant Community

To assess plant community composition and abundance of *M. maximus* within each plot, we surveyed plant species richness and percent foliar cover within two randomly assigned 1 m × 1 m quadrats. Invasion (i.e., invaded or uninvaded) was assigned based on the presence of *M. maximus* in the sampled quadrats. In invaded plots, *M. maximus* was the dominant member of the plant community (mean percent cover = 39.5 ± 17.6%), whereas uninvaded plots had no *M. maximus* present (0% cover) (Appendix S2: Figure S2).

### 2.2 Soil Collection

Our sampling design was hierarchical, with five pooled soil samples collected within each plot and six treatment plots nested within each of three sites. Within each plot, we randomly collected ten soil samples (total samples per site: 60). We removed the top litter layer and collected soil to a depth of 20 cm with a spade wiped clean and sterilized with 70% ethanol between each sample. We then randomly pooled two soil cores to reduce our sample number to five soil samples per plot. The five pooled samples represented independent biological observations collected from different locations within each plot to characterize within‐plot spatial heterogeneity (see *Statistical analysis*). Soil samples were further separated into soil property or microbial analyzes as described in Appendix S1.

### 2.3 Soil Properties

Elemental analysis of carbon (C) and nitrogen (N) was performed via dry combustion using an Elementar (vario EL cube, Elementar Americas). Soil organic C (SOC) was determined via acid fumigation and Active C was measured as permanganate oxidizable C (Weil et al. 2003). Soil pH was measured using a 2:1 water to soil ratio on air‐dried soil. Soil nutrient concentrations were determined by extracting samples with a Mehlich III solution (Mehlich 1984). Soil texture was determined on soil samples composited by sampling site using a (Gee and Bauder 2018) modified hydrometer method (Black et al. 1965) as described in Appendix S1. A high throughput fluorometric assay method (German et al. 2011; A. P. Smith et al. 2015) was used to measure the potential extracellular enzyme activity of three key enzymes involved in nutrient cycling processes: β‐glucosidase, N‐acetylglucosaminidase (NAGase), and phosphatase. Activities were normalized by SOC. Appendix S1.

### 2.4 Library Preparation and Post‐Sequencing Processing

For each sample, we extracted total genomic DNA from approximately 250 mg of soil using the PowerSoil DNA Isolation Kit (Qiagen, USA). Following extraction, we assessed DNA quality using a NanoDrop Lite Spectrometer (Thermo Scientific, USA) and then followed a two‐step process for library preparation (U'ren and Arnold 2017). For the inner PCR, we amplified the regions of interest—the fungal internal transcribed spacer 1 (ITS1) region and the bacterial 16S V4‐V5 region—and added Illumina adapters in triplicate. In the outer PCR, we added sample‐specific barcodes. We included both positive and negative controls through our process as outlined in Appendix S1 along with details on DNA amplification. Samples were submitted to the University of Texas at Austin Genomic Sequencing and Analysis Facility (GSAF) for completion of the outer PCR and sequencing on Illumina MiSeq V2 PE250.

Following sequencing, reads were demultiplexed, and primers were removed from reads at GSAF using standard protocols. We processed raw reads with USEARCH v.10 (Edgar 2010) as described in See Appendix S1.

Taxonomic placement of fungal sequence data was estimated by querying GenBank (Altschul et al. 1990) with validation in UNITE (Kõljalg et al. 2013; Nilsson et al. 2019). Taxonomy of bacterial sequences was assigned using the q2‐feature‐classifier in QIIME 2 (Bolyen et al. 2019) and SILVA 16S rRNA gene database (Quast et al. 2013). The classifier was trained using a naïve Bayes machine‐learning classifier for non‐saline soil (https://zenodo.org/record/6395539) (Bokulich et al. 2018; Kaehler et al. 2019; Robeson et al. 2021).

### 2.5 Statistical Analysis

To account for our hierarchical sampling in which treatment factors were assigned at the plot level with multiple soil samples collected within each plot, we used linear mixed‐effects models that included site and plot nested within site as a random effect (1 | site/plot). This random‐effects structure accounted for the non‐independence of samples collected from the same plot while retaining within‐plot variation in the response variables. For community analysis, we retained multiple pooled soil samples to capture fine‐scale spatial heterogeneity in microbial community composition within plots. Analysis structure is shown in Appendix S2: Table S2.

Due to the bimodal distribution of *M. maximus* cover and designing our study to assess invaded and uninvaded communities, invasion was represented by a categorical variable instead of a quantitative variable in all analyzes. To assess how invasion varied with plant community and disturbance, we used a linear mixed‐effects model assessing percent cover of *M. maximus* with disturbance and vegetation type as fixed effects, and site and plot nested within site as random effects (~ disturbance * invasion + (1 | site/plot); “lmer” command in *lmerTest* package version 3.2–1) (Kuznetsova et al. 2017). We assessed the significance of fixed effects with a Type III ANOVA with Satterthwaite's approximation on the fitted mixed‐effects model. We restricted these analyzes to invaded plots as there was no *M. maximus* in uninvaded plots (Appendix S2: Figure S2a). We assessed plant community richness and Shannon's diversity across our three main factors and their interactions (vegetation type, invasion, and disturbance) as above. We transformed plant diversity with natural log prior to analysis and assessed model residuals for normality. We assessed how soil properties varied across the landscape with a principal component analysis (PCA) (“prcomp” command from the *stats* package version 4.3.1) followed by an ANOVA analysis of the first principal component (PC1) and the second principal component (PC2) as a function of our three main factors and their interaction as fixed effects and site and plot as random factors as above. We included active C, SOC, N, phosphorus (P), magnesium (Mg), manganese (Mn), iron (Fe), potassium (K), calcium (Ca), and pH in the PCA (Appendix S2: Table S3). Prior to running the PCA, the soil data was standardized using z‐scores (“decostand” command from *vegan* package version 2.6.8). We then assessed variation of soil enzymatic activity, as above. The enzymatic data were transformed prior to analysis (see Data accessibility for more details).

We assessed bacterial and fungal OTU richness and diversity with an ANOVA (“lm” command in *stats* package), as above (~ vegetation type * invasion *disturbance + (1 | site/plot)). Model residuals were visually assessed for normality. To assess differences in soil fungal and bacterial community composition as a function of vegetation type, we conducted an analysis of similarity (ANOSIM) with two indices of dissimilarity: a presence‐absence based measure (Jaccard) and an abundance‐based measure (Morisita‐Horn). We limited permutations to site, but not plot to capture fine‐scale spatial heterogeneity in microbial community composition within plots. We assessed each dataset for equal variance (“betadisper” command in *vegan* package) prior to analysis (Oksanen et al. 2026). The bacterial community dataset did not have equal variance for either the Jaccard (F_1,118_ = 9.6, *p* = 0.0025) or the Morisita‐Horn dissimilarity index (F_1,118_ = 10.2, *p* = 0.0018). For the bacterial datasets then, we used the non‐parametric permutational analysis of variance (PERMANOVA; “adonis2” command in *vegan* package) to partition variation due to vegetation type. We visually evaluated differences in soil fungal and bacterial communities using a non‐metric multidimensional scaling (NMDS).

### 2.6 Analyzes Performed Within Each Vegetation Type

To account for collinearity of soil traits, we performed a PCA as above. We assessed correlation of PC1 and PC2 to plant diversity with a mixed effects model, as above. In grasslands, soil PC1 was significantly correlated to plant Shannon's diversity (*t* = 2.29, *p* = 0.0448). To account for this correlation, we ran a generalized linear model with plant Shannon's diversity and soil PC1 as the input variables and used the model residuals to represent plant diversity in subsequent models (hereafter, plant Shannon's residuals). We assessed the correlation of soil enzymes to fungal and bacterial diversity, plant diversity, and soil PC1 with a linear mixed effects model (Appendix S2: Table S2). We used Fisher's alpha diversity for the microbial communities, as it is robust to communities with many rare species and a few common species (i.e., follows a log‐series distribution). We assessed fungal and bacterial OTU richness and diversity as a function of plant diversity and soil components, with a mixed effects model as above (Appendix S2: Table S2). Based on results from the soil analyzes (see Results), we used soil PC1 for grasslands and soil PC2 for woody patches in analyzes of microbial diversity. For all analyzes, model residuals were assessed for fit.

We assessed community variation of fungi and bacteria as a function of plant diversity, soil traits, and geographical distance (Appendix S2: Table S2) with permutations constrained to sites. Geographical distance was included to account for spatial autocorrelation. We created a Euclidean‐based distance matrix of site latitude and longitude and then computed a principal coordinates of neighborhood matrix to transform spatial distances to rectangular data (“pcnm” command in *vegan* package) (Dray et al. 2006; Legendre and Legendre 2012).

Based on results within each vegetation type, we conducted structural equation modeling (SEM) for each system to assess both direct and indirect effects (“sem” command in *lavaan* package) (Rosseel 2012). The best SEM models were selected based on the corrected Akaike (AICc) information criterion, model Chi‐squared, Root Mean Square Error of Approximation (RMSEA), and the Comparative Fit Index (CFI). We used geographical distance and plant Shannon's diversity as exogenous variables but used distinct models for each vegetation type based on our results. The fungal and bacterial communities were represented by the first axis from NMDS based on Jaccard and Morisita‐Horn dissimilarities, respectively. This axis was selected because it captured meaningful ecological structure (see Results) and provided a continuous variable suitable for SEM. Alternative approaches (e.g., PCoA) explained < 11% of the variance and were not retained. For the full model, see Data availability.

### 2.7 Taxonomic Analyzes

We assessed differences in fungal and bacterial relative OTU abundance of taxonomic groups across grasslands and woody patches with chi‐square tests. For fungi, we ran analyzes at the phylum level followed by finer scale analyzes within each phylum. These included class level within Ascomycota and order level analyzes of Agaricomycetes, which composed about 90% of the Basidiomycota community. We assessed differences in OTU richness and relative OTU abundance of genera within the subphylum Glomeromycotina (Phylum: Mucoromycota), which contains arbuscular mycorrhizal (AM) fungi with a non‐parametric Kruskal‐Wallis rank sum test and *t*‐test, respectively. For bacterial communities, we focused our analyzes at the phylum level with a class level analysis of Actinobacteriota. We assessed differences in cyanobacteria between grasslands and woody patches, analyzing taxonomic differences at the family level with a chi‐square test and OTU richness with a Kruskal‐Wallis test.

## 3 Results

We addressed our questions using an integrative, theme‐based structure rather than addressing each question in isolation. Because the environmental, soil, and plant variables used in the models underpin all subsequent microbial analyzes, we first described key abiotic and biotic properties and then presented microbial responses. Individual analyzes speak to one or more of the stated questions but are organized by major components of the conceptual and methodological design.

**FIGURE 1:**
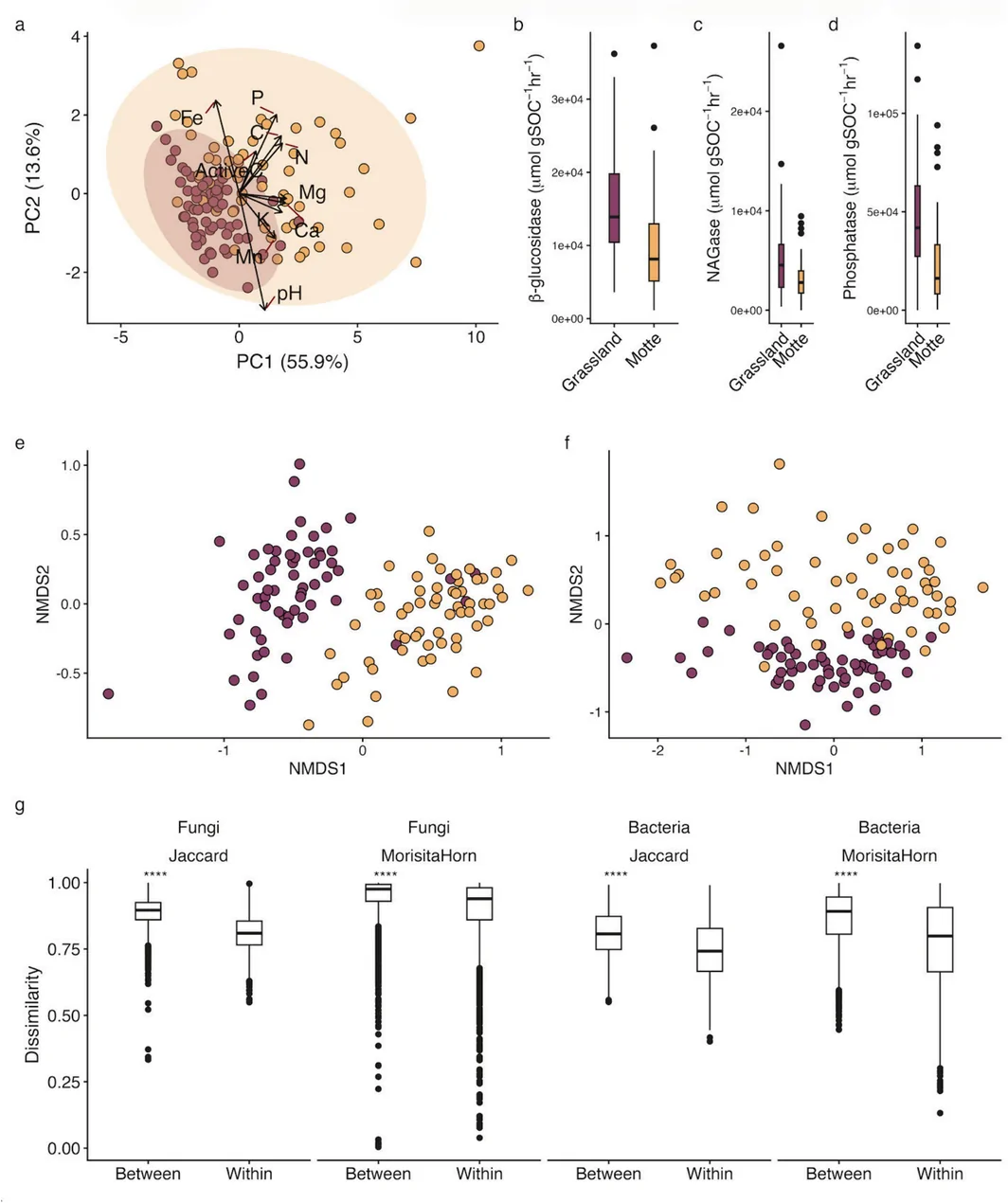
Vegetation type explained the most variation across the soil environment. The nutrient poor (a) soil in grasslands was correlated to higher enzymatic activity (b to d). Fungal and bacterial communities were distinct in grasslands and woody patches (e, Jaccard dissimilarity index; f, Morisita‐Horn dissimilarity index). Overall, communities of fungi and bacteria were more similar to each other within each vegetation type (“Within”) than between vegetation types (“Between”) (g). Asterisks indicate a *p*‐value of < 0.0001. Panel e: Stress = 0.14; panel f: Stress = 0.14.

Within invaded sites, disturbance and invasion did not affect *M. maximus* cover, although cover was 14.7% higher in grasslands (ANOVA, F_1,18_ = 4.05, *p* = 0.0790; Appendix S2: Figure S2b). Plant species richness and diversity were higher in grasslands and lower in plots invaded by *M. maximus* (Appendix S2: Table S4; Appendix S2: Figure S3). The soil textural class was characterized as sandy loam across the sampled area (% clay = 16.3 ± 1.0 std. dev.; % sand = 75.7 ± 1.0; % silt = 8.0 ± 1.0). Soil traits such as nutrients (PC1 and PC2) and enzymatic activity differed significantly between grasslands and woody patches, with grasslands being more nutrient‐poor but demonstrating higher levels of enzymatic activity (Table 1; Figure 1a–d). To a lesser extent, soil nutrient availability (PC1) increased with mechanical disturbance (Appendix S2: Figure S4). The mean and standard deviation of soil chemical, physical, and enzymatic properties by sampling site are included in Appendix S2: Table S2a,b.

**TABLE 1:** Vegetation type explained more variation in all soil traits than did invasion and disturbance.

	Vegetation type	Invasion	Disturbance	Veg. type × Inv.	Veg. type × Dist.	Inv. × Dist.	Veg. type × Inv. × Dist.
PC1 of soil traits							
F‐value	17.5	3.3	11.9	0.4	1.7	0.9	0.0
*p*	**0.0007**	0.0884	**0.0033**	0.5612	0.2061	0.3600	0.8741
PC2 of soil traits							
F‐value	7.7	0.0	2.7	2.8	0.2	1.2	2.7
*p*	**0.0149**	0.9878	0.1257	0.1155	0.6361	0.2863	0.1203
β‐galactosidase							
F‐value	15.6	0.2	2.4	2.1	0.3	0.0	0.2
*p*	**0.0014**	0.6439	0.1467	0.1687	0.6016	0.9984	0.6811
N‐acetyl‐β‐glucosaminidase							
F‐value	6.3	0.4	0.2	0.5	0.0	0.3	0.2
*p*	**0.0249**	0.5591	0.6705	0.4926	0.9534	0.5834	0.6465
Phosphatase							
F‐value	25.8	0.2	2.5	0.0	0.7	2.5	0.4
*p*	**< 0.0001**	0.6425	0.1190	0.8854	0.4173	0.1193	0.5333

*Note:* Values in boldface indicate significant differences. Principle coordinate axes 1 and 2 (PC1 and PC2, respectively) are composed of active C, SOC, N, P, Mg, Mn, Fe, K, Ca, and pH.

In total, we isolated 1351 fungal and 5664 bacteria OTUs across all our sites. Fungal OTU richness and diversity did not vary significantly across invasion, plant community, or disturbance (Table 2). Average fungal OTU richness and diversity across sites were 178 ± 42 and 45.5 ± 11.3, respectively. Bacterial OTU richness and diversity across all sites averaged 2861 ± 847 species and 2230.0 ± 661.0, respectively. There was an interactive effect of invasion and disturbance on OTU richness of bacteria, with richness highest in uninvaded sites without recent mechanical disturbance (Table 2; Appendix S2: Figure S5a). Bacterial diversity was higher in grasslands (Table 2; Appendix S2: Figure S5b).

**TABLE 2:** OTU richness and diversity of soil fungal and bacterial communities across our main factors and all sites.

	Fungi				Bacteria			
	OTU richness		Diversity		OTU richness		Diversity	
	*F*‐value	*p*	*F*‐value	*p*	*F*‐value	*p*	*F*‐value	*p*
Vegetation type	0.10	0.7535	0.21	0.6572	3.42	0.0828	12.21	**0.0036**
Invasion	0.72	0.4096	0.36	0.5596	0.88	0.3623	0.26	0.6180
Disturbance	1.44	0.2501	0.83	0.3771	1.28	0.2737	1.80	0.2010
Veg. type × Inv.	3.29	0.0914	2.43	0.1411	0.20	0.6549	0.86	0.3686
Veg. type × Dist.	0.05	0.8291	0.25	0.6257	0.50	0.4881	0.55	0.4684
Inv. × Dist.	1.77	0.2043	1.33	0.2679	9.34	**0.0075**	4.00	0.0653
Veg. type × Inv. × Dist.	0.52	0.4822	0.17	0.6859	0.02	0.8821	0.24	0.6300

*Note:* Values in boldface indicate significant differences.

Grasslands and woody patches contained distinct fungal and bacterial communities (Figure 1e,f). Communities were more similar to those found within each vegetation type than between grasslands and woody patches (Figure 1g). In fungal communities, more variation was explained by the presence/absence of OTUs (Jaccard: *p* = 0.0010, *R* ^2^ = 54.0%; Figure 1e) than by their relative abundance (Morisita‐Horn: *p* = 0.0010, *R* ^2^ = 31.1%; Appendix S2: Figure S5c). Conversely, bacterial communities showed the opposite pattern, with more variation explained by the relative abundance of OTUs (Morisita‐Horn: F_1,119_ = 13.7, *p* = 0.0050, *R* ^2^ = 10.4%; Figure 1f) than by the presence/absence of OTUs alone (Jaccard: F_1,119_ = 7.0, *p* = 0.0050, *R* ^2^ = 5.6%; Appendix S2: Figure S5d).

### 3.1 Grasslands

**FIGURE 2:**
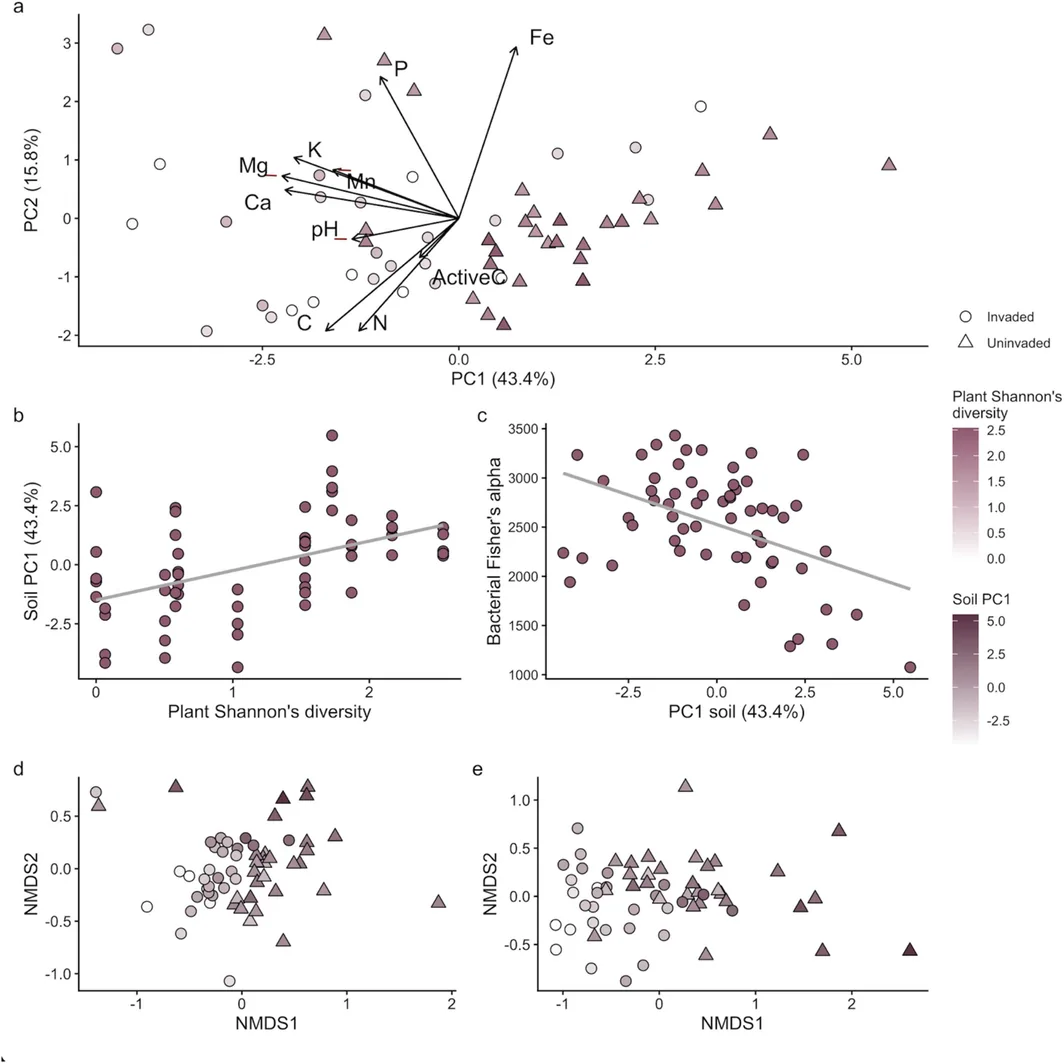
In grasslands, soil properties in invaded and uninvaded areas were significantly different (a). Plant diversity was negatively correlated with soil nutrient availability (b). Bacterial diversity was positively correlated with soil nutrient availability (c). Both microbial communities showed strong separation along the PC1 soil gradient and invasion (d: Fungi, Jaccard index, stress = 0.16; e: Bacteria, Morisita‐Horn index, stress = 0.13).

Soil PC1 and PC2 explained 43.4% and 15.8%, respectively, of the variation in grassland soils (Figure 2a). In the soil analysis, approximately 26.8% of soil PC1 (adjusted *R* ^2^ = 24.2%, F_2,56_ = 10.2, *p* = 0.0002) was explained by our model with PC1 positively correlated with Plant Shannon's residuals (Figure 2b; *t* = 4.2, *p* < 0.0001). Soil PC2 and soil enzymes were not correlated with the explanatory variables in our models. Bacterial Fisher's alpha was negatively correlated with soil PC1 (Figure 2c; *t* = −2.4, *p* = 0.0243). Fungal Fisher's alpha and bacterial OTU richness were not significantly correlated with the variables included in our model.

Soil properties and plant diversity explained a total of 5.6% of variation in OTU presence or absence (Figure 2d) and 6.3% of OTU abundance (Appendix S2: Figure S6a; Table 3). In bacterial communities, more variation was explained by soil traits than plant diversity, and overall, our model explained 7.8% of variation in OTU presence or absence (Appendix S2: Figure S6c) and 13.4% of OTU abundance (Figure 2e; Table 3). Neither fungal nor bacterial communities were significantly correlated with geographic distance (Table 3).

**TABLE 3:** PERMANOVA of fungal and bacterial communities within grasslands and woody patches.

	Grassland											
	Fungi						Bacteria					
	Jaccard			Morisita‐Horn			Jaccard			Morisita‐Horn		
	*F*‐value	*p*	*R* ^2^	*F*‐value	*p*	*R* ^2^	*F*‐value	*p*	*R* ^2^	*F*‐value	*p*	*R* ^2^
Soil PC1	2.0	**0.0050**	3.26	2.1	**0.0050**	3.50	5.0	**0.0050**	7.78	9.8	**0.0050**	13.36
Plant Shannon's diversity	1.6	**0.0050**	2.60	1.7	**0.0050**	2.77	1.3	0.0700	N.S.	2.0	0.0600	N.S.
Geographical distance	1.6	0.5550	N.S.	1.5	0.6850	N.S.	3.0	0.6100	N.S.	6.4	0.0650	N.S.
Soil PC1 × plant div.	1.0	0.3800	N.S.	0.7	0.8750	N.S.	1.1	0.3550	N.S.	1.0	0.4550	N.S.
Total			**5.85**			**6.27**			**7.78**			**13.36**

	Woody patches											
	Fungi						Bacteria					
	Jaccard			Morisita‐Horn			Jaccard			Morisita‐Horn		
	*F*‐value	*p*	*R* ^2^	*F*‐value	*p*	*R* ^2^	*F*‐value	*p*	*R* ^2^	*F*‐value	*p*	*R* ^2^
Soil PC1	3.3	**0.0050**	5.39	4.0	**0.0050**	6.33	6.7	**0.0050**	10.25	12.7	**0.0050**	17.36
Plant Shannon's diversity	1.8	**0.0050**	2.96	1.9	**0.0050**	3.06	2.1	**0.0050**	3.21	3.2	**0.0050**	4.47
Geographical distance	1.4	0.0900	N.S.	1.6	0.3450	N.S.	1.4	**0.0550**	2.12	2.00	**0.0400**	2.72
Soil PC1 × plant div.	1.1	0.2200	N.S.	1.1	0.2700	N.S.	1.2	0.1400	N.S.	1.3	0.1550	N.S.
Total			**8.35**			**9.39**			**15.58**			**24.55**

*Note:* Values in boldface indicate significant differences. Total variation explained includes only variation for the significant variables and is given in percentage.

**FIGURE 3:**
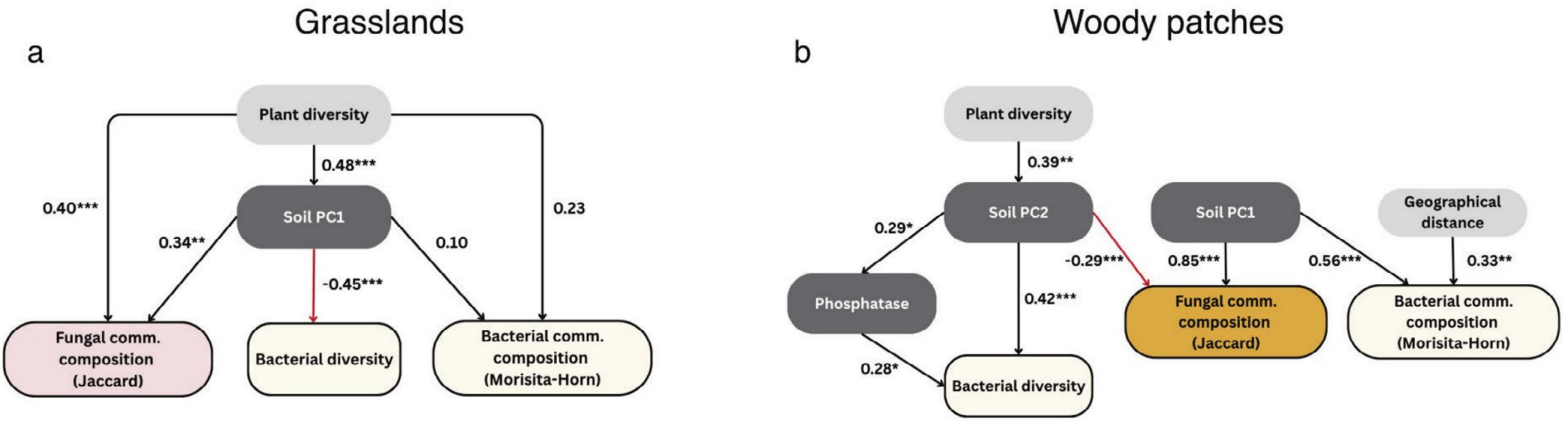
Structural equation modeling for grasslands (a) and woody patches (b). Numbers on arrows are standardized path coefficients, representing the direction and strength of relationships with larger values indicating stronger relationships. Black arrows denote positive relationships and red arrows negative relationships. All variables were measured (observed) rather than latent constructs. *** *p* < 0.001, ** *p* < 0.01, * *p* < 0.05.

The SEM model (Figure 3a) we used had an excellent fit (χ^2^ = 0.118, df = 1, *p* = 0.731; CFI = 1.00; TLI = 1.00; RMSEA < 0.001; SRMR = 0.010; *n* = 59). Plant diversity was positively associated with the soil chemistry gradient in grasslands (soil PC1; β = 0.478, *p* < 0.0010), which in turn was negatively associated with bacterial Fisher's alpha (β = −0.448, *p* < 0.0010). Consequently, plant diversity had a significant indirect negative effect on bacterial diversity through soil chemistry (β = −0.214, *p* < 0.0010). Fungal community composition was positively associated with both soil PC1 (β = 0.340, *p* = 0.0030) and plant diversity (β = 0.403, *p* < 0.0010). In contrast, bacterial community composition was not significantly associated with either soil PC1 or plant diversity. After accounting for plant diversity and soil chemistry, fungal and bacterial community composition remained positively correlated (residual covariance β = 0.618, *p* < 0.0010), suggesting that additional unmeasured factors influenced both microbial communities. The model explained 22.9% of the variation in soil PC1, 20.1% of bacterial Fisher's alpha, 41.0% of fungal community composition, and 8.5% of bacterial community composition.

### 3.2 Woody Patches

**FIGURE 4:**
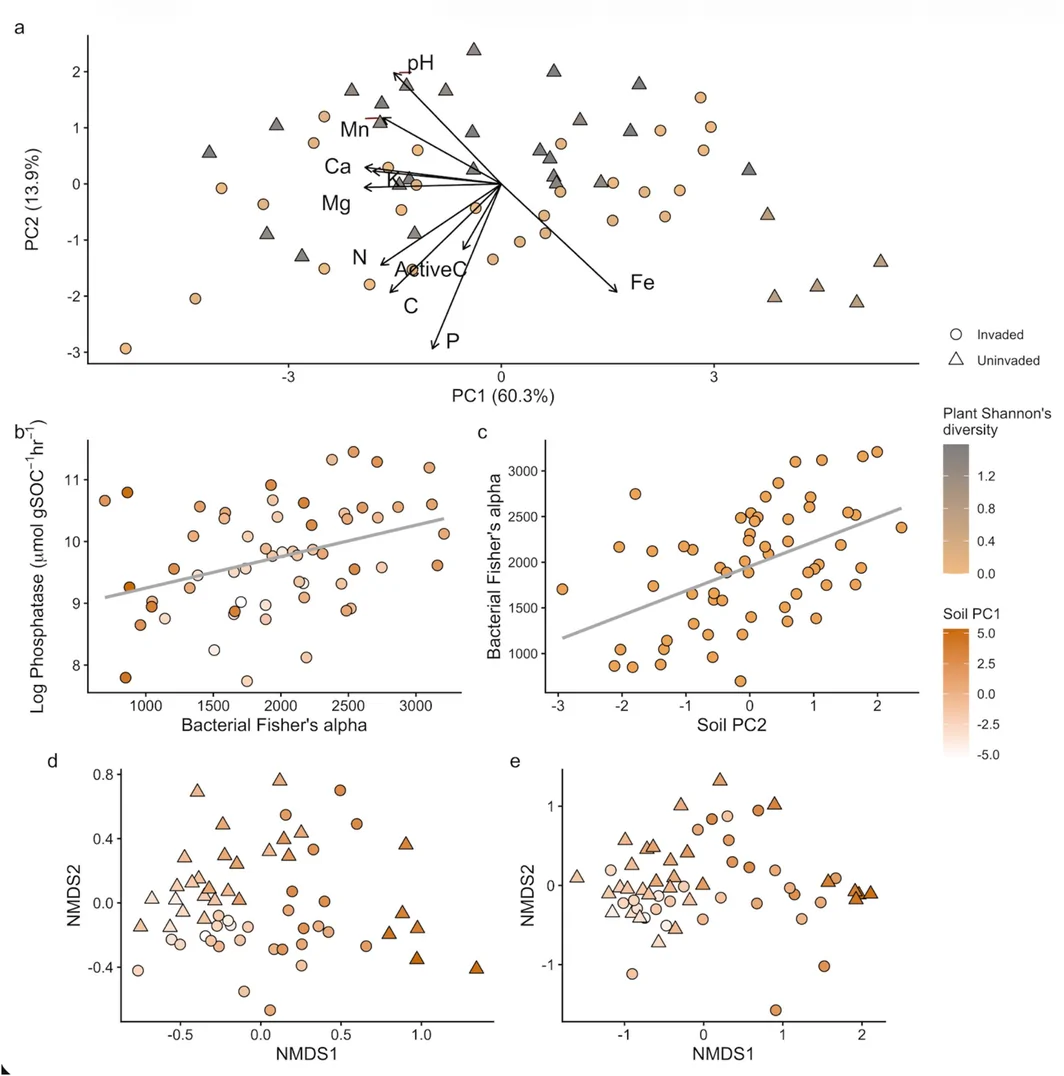
In woody patches, there was a greater overlap of soil properties of invaded and uninvaded area within uninvaded areas having higher overall plant Shannon's diversity (a). Phosphatase abundance was positively correlated with bacterial diversity and soil PC1 (b). PC2 was positively correlated with bacterial diversity (c). Both microbial communities were structured by PC1 soil and invasion (d: Fungi, Jaccard index, stress = 0.18; e: Bacteria, Morisita‐Horn index, stress = 0.15).

Soil PC1 and PC2 explained 60.3% and 13.9%, respectively, of the variation in soil traits associated with woody patches (Figure 4a). Beta‐glucosidase was positively correlated with bacterial diversity (*t* = 2.0, *p* = 0.0471). Phosphatase was positively correlated with bacterial diversity (Figure 4b; *t* = 2.8, *p* = 0.0081) and soil PC1 (*t* = 3.2, *p* = 0.0022).

Fungal OTU richness and Fisher's alpha were not significantly correlated with our model. Bacterial Fisher's alpha was positively correlated with soil PC2 (Figure 4c; *t* = 3.6, *p* = 0.0008). Soil traits explained the most variation in fungal and bacterial communities of woody patches, followed by plant diversity (Table 3). In fungal communities, our model explained a total of 8.4% of variation in OTU presence or absence (Figure 4d) and 9.4% of OTU abundance (Appendix S2: Figure S6b; Table 3). In bacterial communities, our model explained 15.6% of variation in OTU presence or absence (Appendix S2: Figure S6d) and 24.6% of OTU abundance (Figure 4e; Table 3).

The SEM provided a moderate representation of the covariance structure of the woody patch data (χ^2^ = 31.51, df = 14, *p* = 0.005; CFI = 0.904; TLI = 0.828; RMSEA = 0.146; SRMR = 0.0.89; *n* = 59). Although overall model fit was weaker than for grasslands (Figure 3b), the retained pathways were selected a priori based on mixed‐model and community analyzes, and no substantial omitted pathways were identified (all modification indices < 10). Plant diversity was positively associated with soil PC2 (β = 0.391, *p* = 0.0010). Soil PC2 was positively associated with phosphatase activity (β = 0.287, *p* = 0.0220), and both phosphatase activity (β = 0.276, *p* = 0.0170) and soil PC2 (β = 0.422, *p* < 0.0010) were positively associated with bacterial Fisher's alpha. Although soil PC2 exhibited a positive indirect effect on bacterial diversity through phosphatase activity, this mediation pathway was not statistically significant. Fungal community composition was strongly associated with both soil PC1 (β = 0.852, *p* < 0.0010) and soil PC2 (β = −0.289, *p* < 0.0010), whereas bacterial community composition was positively associated with soil PC1 (β = 0.590, *p* < 0.0010) and geographic distance (β = 0.346, *p* = 0.0010). After accounting for soil properties and geographic distance, fungal and bacterial community composition remained positively correlated (residual covariance β = 0.466, *p* = 0.002). The model explained 15.3% of the variation in soil PC2, 8.2% of phosphatase activity, 32.0% of bacterial Fisher's alpha, 81.0% of fungal community composition, and 41.6% in bacterial community composition.

### 3.3 Taxonomic Analyzes

**FIGURE 5:**
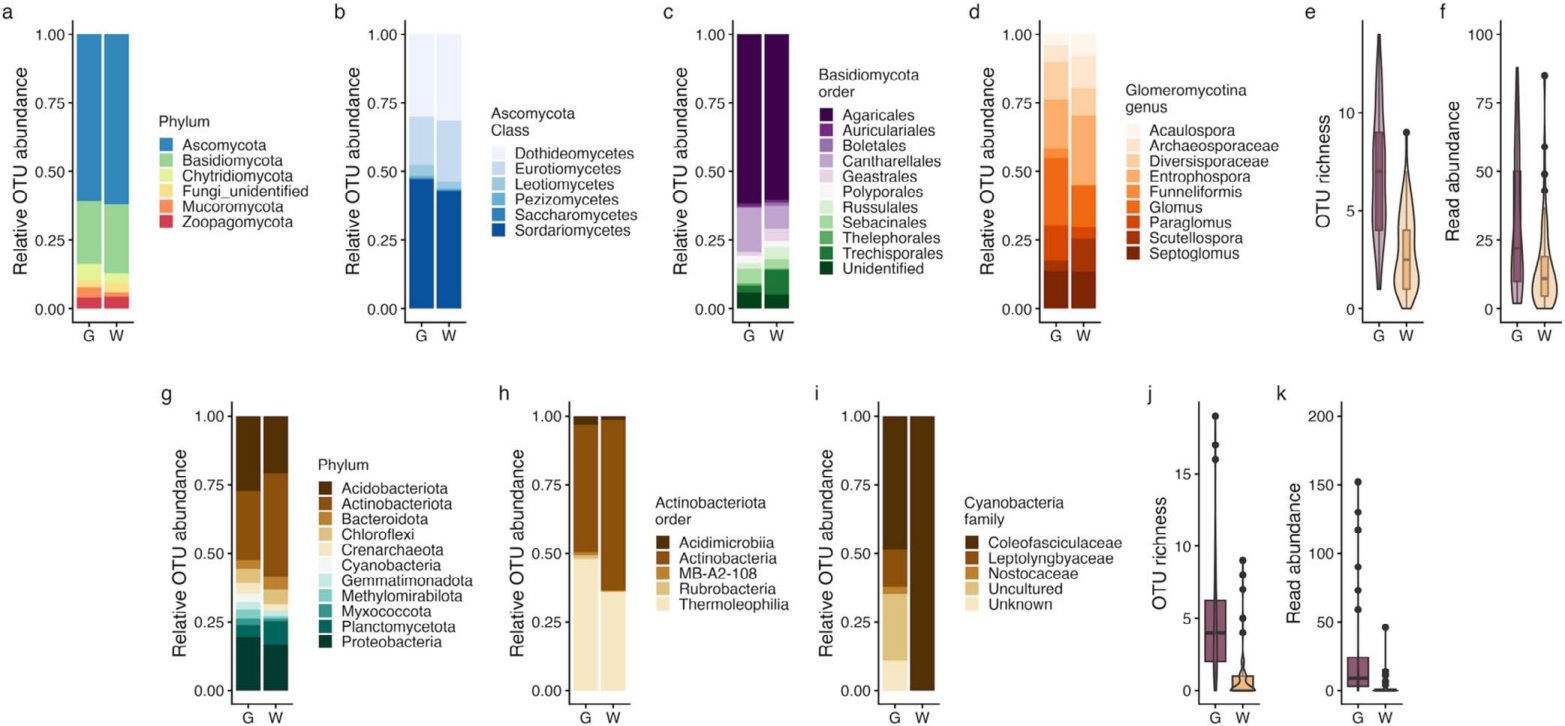
Relative abundance of fungal and bacterial OTUs varied between grasslands and woody patches at the level of phylum (a, g), at the class level within Ascomycota (b), and at the order level in Basidiomycota (c) and Actinobacteriota (h). The relative OTU abundance of arbuscular mycorrhizal fungal genera (d) and cyanobacterial genera (i) varied between grasslands and woodlands, with grasslands having higher OTU richness (e, j) and abundance (f, k) than woodlands. G = grassland, W = woody patch.

There were large differences in the relative OTU abundance of taxonomic groups in woody patches and grasslands (Figure 5). Although the same fungal phyla were present in both grasslands and woody patches, the relative OTU abundance differed ($\chi_{5}^{2}$ = 117.2, *p* < 0.0001) with Mucoromycota and Chytridiomycota more abundant in grasslands and Basidiomycota more abundant in woody patches (Figure 5a). Ascomycetes at the class level (Figure 5b, $\chi_{5}^{2}$ = 51.1, *p* < 0.0001) and basidiomycetes at the order level (Figure 5c, $\chi_{11}^{2}$ = 151.7, *p* < 0.0001) differed between the two vegetation types. Sordariomycetes, Cantharellales, and Sebacinales were more abundant in grasslands, whereas Dothideomycetes, Eurotiomycetes, Trechisporales, Russulales, and Geastrales were more prominent in woody patches. There were strong differences in the relative abundance of genera belonging to Glomeromycotina, the subphylum of arbuscular mycorrhizal (AM) fungi (Figure 5d, $\chi_{10}^{2}$ = 31.1, *p* = 0.0006). Overall, AM fungi were more OTU rich and abundant in grasslands (Figure 5e, Kruskal $\chi_{1}^{2}$ = 43.1, *p* < 0.0001; Figure 5f, *t*_117.5_ = 4.4, *p* < 0.0001).

The relative OTU abundance of bacterial phyla (Figure 5g, $\chi_{15}^{2}$ = 509.7, *p* < 0.0001) and orders within the class Actinobacteriota (Figure 5h, $\chi_{4}^{2}$ = 293.8, *p* < 0.0001) differed between grasslands and woody patches. Distinct families of Cyanobacteria were present in grasslands compared to woody patches (Figure 5i, $\chi_{5}^{2}$ = 78.8, *p* < 0.0001) with higher OTU richness (Figure 5j, Kruskal $\chi_{1}^{2}$ = 48.9, *p* < 0.0001) and relative OTU abundance (Figure 5k, Kruskal $\chi_{1}^{2}$ = 43.9, *p* < 0.0001) in grasslands.

## 4 Discussion

Savannas are defined by the coexistence of grasslands and woodlands, creating a heterogeneous landscape. We showed that the aboveground heterogeneity of biphasic savannas is reflected belowground with the grassland matrix and woody patches hosting taxonomically and functionally distinct microbial communities and soil environments (Figure 1). Vegetation type and soil nutrient availability were strongly associated with microbial community composition and function. These findings align with long‐standing evidence that plant communities within savannas structure soil properties and feedbacks (Sankaran et al. 2004; Scholes and Archer 1997), but we can now identify correlative links between these processes and the soil microbial communities (Figure 3). The mechanism of coexistence of a biphasic system in savannas has been a long‐standing question (Scholes and Archer 1997). Our study highlights that the soil microbial community should be considered in coexistence models as they provide an added dynamic and possible mechanism for understanding shifts or maintenance in aboveground plant communities over time (Bever et al. 2010).

Despite a shared climate and close proximity, grasslands and woody patches supported distinct microbial community assembly processes. Grasslands exhibited a relatively simple structure in which plant diversity was tightly coupled to soil nutrient availability, and microbial communities were relatively homogeneous across space, showing little effect of geographic distance. Lower soil nutrient availability in grasslands was correlated with higher enzymatic activity as well as more diverse AM fungi and cyanobacteria—groups that promote nutrient acquisition and primary productivity under low‐resource conditions (in't Zandt et al. 2019; Rodríguez‐Caballero et al. 2018). At low levels of soil nutrient availability, AM fungi can help retain soil nutrients, but this effect disappears as soil nutrient availability increases, such as after disturbances or invasion of non‐native plants (Inderjit and van der Putten 2010; Qiu et al. 2022; van der Heijden 2010). In contrast, woody patches displayed a more complex interaction among plant diversity, soil properties, and spatial autocorrelation, with microbial communities more strongly associated with variation in soil chemistry than in grasslands. Bacterial diversity was positively correlated with cellulose degradation and conversation of phosphorus into usable inorganic phosphate. Woody patches were also enriched in fungal taxa specializing in lignocellulose degradation aligning with woody litter inputs reflecting the turnover in plant guilds (Floudas et al. 2012). Together, these differences illustrate how savanna mosaics support distinct microbial niches aligned with aboveground vegetation and resource regimes. These differences highlight the importance of spatially explicit processes in savanna mosaics, although it is unclear whether soil microbial communities may act as stabilizers or, if perturbed, facilitate directional shifts in plant community through feedback dynamics (Bever et al. 2010).

As microbial composition was highly sensitive to plant and soil drivers in both vegetation types, invasion could disrupt coexistence and alter belowground processes in ways that could reinforce the dominance of invasive grasses (Harpole et al. 2016). With *M. maximus* invasion, we have previously seen a shift from a predominantly negative plant–soil feedback pattern in grasslands that supports coexistence and diversity to positive plant–soil feedback promoting the establishment and growth of *M. maximus* (Bowman et al. 2023). These shifts in plant–soil feedback correspond to an increase in soil nutrient availability and input of litter from *M. maximus* that is rich in secondary metabolites which inhibit germination and growth of some native plant species (Morrison et al. 2023). Disturbance had a similar, although weaker, effect, but its additive negative impact on diversity suggests that interacting disturbances could exacerbate long‐term effects on these communities. Because microbes appear to track these drivers so closely, invasion has the potential to destabilize the coexistence mechanisms that maintain savanna mosaics, weaken negative plant–soil feedback in grasslands, and alter decomposer networks in woody patches. Together, these findings highlight how above–belowground linkages underpin savanna resilience and reveal the vulnerability of these systems when feedbacks are disrupted by invasive grasses and multiple stressors.

A parallel could be drawn between woody plant encroachment into grasslands and encroachment by *M. maximus* . The effect of invasion by *M. maximus* was significant, with a decrease in plant diversity, an increase in soil nutrient availability, and a corresponding shift in soil microbial communities. With both invasion and woody plant encroachment, plant litter and root exudates shift, which is ultimately followed by an increase in soil nutrient availability and a change in the taxonomic and functional composition of the soil microbial community (Archer 1995). Different litter types and root exudate profiles can fundamentally reshape microbial assemblages, potentially driving succession from grass‐dominated to woody states. The persistence of grasslands may be reinforced by low nutrient availability and microbial community composition providing a type of biotic resistance (Chung et al. 2019).

Taken together, these results suggest that savanna mosaics are structured by tight linkages between vegetation, soil nutrients, and microbial communities. Grasslands and woody patches foster distinct microbial assemblages that reflect differences in nutrient regimes, plant functional groups, and resource inputs, while invasion threatens to disrupt these couplings. This study highlights how aboveground–belowground feedbacks contribute to the resilience and vulnerability of savanna ecosystems, with implications for their management under ongoing invasion and climate change.

## Author Contributions

**Elizabeth A. Bowman:** data curation (lead), formal analysis (lead), investigation (lead), methodology (equal), supervision (equal), visualization (lead), writing – original draft (lead), writing – review and editing (equal). **A. Peyton Smith:** data curation (supporting), formal analysis (supporting), investigation (supporting), methodology (equal), writing – review and editing (equal). **Aaron C. Rhodes:** conceptualization (equal), writing – review and editing (equal). **Robert M. Plowes:** conceptualization (equal), funding acquisition (equal), project administration (equal), writing – review and editing (equal). **Lawrence E. Gilbert:** conceptualization (equal), funding acquisition (equal), project administration (equal), writing – review and editing (equal).

## Funding

This study was funded by the Lee and Ramona Bass Foundation.

## Conflicts of Interest

The authors declare no conflicts of interest.

## Supporting information


**Appendix S1:** Supplemental methods.

**Appendix S2:** Supplemental Figures and Tables.
**Figure S1:** Mean annual precipitation (MAP) varied 6.7 mm across sampling sites.
**Figure S2:** Histogram of percent cover of *M. maximus* across all sites (a) and percent cover as a function of vegetation type in invaded sites (b). In panel a, each sample is colored by its invasion status (white = uninvaded; blue‐green = invaded).
**Figure S3:** Plant species richness and diversity were higher in grasslands (a) and uninvaded sites (b). Here, only plant species richness is shown.
**Figure S4:** PCA of soil traits as a function of invasion (a) and disturbance (b). Boxplot of PC1 as a function of vegetation type (c), invasion (d), and disturbance (e). The point colors match those in the corresponding boxplots. Panel a colors match those in panel d; panel b colors match those in panel e.
**Figure S5:** Invasion and disturbance had an interactive effect on bacterial OTU richness (a). Bacterial diversity was higher in grasslands (b) NMDS of fungal (c; stress = 0.23) and bacterial (d; stress = 0.12) communities across all sites. The dissimilarity indices represented here are Morisita‐Horn and Jaccard, respectively.
**Figure S6:** NMDS of fungal communities (a, b) and bacterial communities (c, d) in grasslands (a, c) and woody patches (b, d). The dissimilarity index used in panels a (stress = 0.23) and b (stress = 0.26) is the Morisita‐Horn and the Jaccard in panels c (stress = 0.12) and d (stress = 0.11).
**Table S1:** List of sampling sites and associated metadata. Climate data was downloaded from WorldClim (Fick and Hijmans 2017). Disturbance year is the most recent brush management event.
**Table S2:** List of analyses with the response variable, fixed effects, and random effects that were included in the models. Notes indicates any transformations or filtering of the data for the analysis.
**Table S3:** Mean and standard error of soil enzymatic and chemical properties (A). Mean and standard error of soil nutrients (B).
**Table S4:** Plant species richness and diversity were higher in uninvaded sites and grasslands.

## Data Availability

The datasets generated during and/or analyzed during the current study are available in the MesquiteSavanna_SoilMicrobialCommunity repository on GitHub (https://doi.org/10.5281/zenodo.17915548). Illumina data are archived at GenBank under BioProject PRJNA1373218: Biphasic savanna paralleled in belowground soil abiotic and biotic environment.
