# Supplementary material for: Soil Microbial Assembly Mirrors a Savanna Mosaic Amid Plant Invasion and Soil Disturbance
Source: Ecol Evol. 2026 Sep 3;16(9):e74284. doi: 10.1002/ece3.74284 (PMC13540272; doi:10.1002/ece3.74284)
Supplement: Supplementary file 1 [file ECE3-16-e74284-s001.docx]

**Appendix S1: Supplemental Methods**

We sampled across three sites in Kenedy and Brooks County, Texas, spanning 33 km2, with distances between sites ranging from 6 to 10 km, on a ranch with documented fire, grazing, and brush management practices. All sites were selected to be in the same soil series, the Palobia loamy fine sand described as a fine-loamy, mixed, active, hyperthemic Typic Natrustalf (Soil Survey Staff et al. 2024). Within each site, we collected soil samples and plant assemblage data from 100 m^2^ plots for each cross (2 disturbance types x 2 invasion types x 2 vegetation types; Supplementary Table S1). In woody patches, we sampled soil from the interior and edges of the patch to capture variation across these areas. To assess disturbance, we sampled from sites with either recent or historical disturbance. Recent disturbances, occurring in the late 1990s to early 2000s, were root-plowing events that included the removal of trees and shrubs with their roots and the complete turnover of the soil (Supplementary Table S1). Old disturbance sites have not been disturbed since 1980, although our information prior to that time is limited. Based on aerial photos from the Texas Geographic Information System (TxGIO), site 2 has not been disturbed since 1943 (Supplementary Table S1) (Texas Geographic Information Office 2022).

Temperature, precipitation, and plant available moisture have been shown to act at broader scales, unlike soil nutrients, which act at a finer scale (Mishra and Young 2020). Our sampling was within a small geographic area with low variation in precipitation and temperature over the sampling area (Supplementary Table S1).

*Plant community*

To assess plant community composition and abundance of Guinea grass within each plot, we surveyed plant species richness and percent foliar cover within two randomly assigned 1 m x 1 m quadrats. Invasion (i.e., invaded or uninvaded) was assigned based on the presence of Guinea grass in the sampled quadrats. In invaded plots, Guinea grass was the dominant member of the plant community (mean percent cover = 39.5 ± 17.6%), whereas uninvaded plots had no Guinea grass present (0% cover) (Supplementary Fig. S1).

*Soil collection*

Within each plot, we randomly collected 10 soil samples (total per site: 60). We removed the top litter layer and collected soil to a depth of 20 cm with a spade, wiping it clean and sterilizing it with 70% ethanol between each sample. We transported soil cores to the lab on ice. We then randomly pooled two soil cores to reduce the sample number to 5 soil samples per plot. The choice of 5 samples per plot was based on OTU accumulation curves from previous sampling efforts. Soil was then subsampled for two pathways: 1) analysis of soil characteristics (nutrients, enzymatic activity, and texture) and 2) metabarcoding of fungal and bacterial communities followed by short-read sequencing on Illumina MiSeq. For metabarcoding, we collected two sets of approximately 250 mg from each pooled soil sample and stored them at -20℃ until extraction. The remaining pooled soil samples were kept on ice in a cooler until they were processed for analysis of soil characteristics.

*Soil chemical properties*

Elemental analysis of carbon (C), nitrogen (N), sulfur (S), and hydrogen (H) was performed via dry combustion using an Elementar (vario EL cube, Elementar Americas). Total soil organic carbon (SOC) was determined following acid fumigation to remove inorganic forms of C (Harris et al. 2001, Dhillon et al. 2015). Dried and fine-ground subsamples were placed in a desiccator and exposed to 12 M HCl vapors for 24 hours to remove carbonates. Following fumigation, samples were dried at 40ºC to remove residual moisture and acid vapors and quantified using dry combustion (as described above). Active C, i.e., permanganate oxidizable carbon (POxC), was used as a proxy to determine the biologically active fraction of SOC based on (Weil et al. 2003) and (Culman et al. 2012). Briefly, 2.50 ± 0.05 g of oven-dried soil (65ºC) was mixed with 0.02 mol L^-1^ KMnO_4_. Tubes were shaken for 2 minutes using a vortex at 240 oscillations per minute, rested in the dark for 10 minutes, and then diluted to a 1% solution. Absorbance on the dilute solution was determined using a Microplate reader (Synergy, Biotek), and absorbance values were calculated into mg kg^-1^ using the following equation:

$POxC=\left( 0.02 mol L^{-1}-\left( a+b \times Abs \right)) \times(9000 mg C mol^{-1} \right)\times\left( 0.02 L solution \div Wt \right)$,

where *0.02 mol L^-1^* is the initial concentration of the KMnO_4_ solution, *a* is the intercept of the standard curve, *b* is the slope of the standard curve, *Abs* is the absorbance of the unknown soil sample, *9000 mg* is the amount of C oxidized by 1 mol of MnO_4_ with Mn^7+^ getting reduced to Mn^4+^, *0.02 L* is the volume of KMnO_4_ solution reacted with soil, and *Wt* is the amount of soil (kg) used in the reaction.

Soil pH was measured using a 2:1 water-to-soil ratio on air-dried soil. Soil nutrient concentrations were determined by extracting samples with a Mehlich III solution (Mehlich 1984), representing plant and microbial available nutrients, and analyzing the extract via Inductively Coupled Plasma Optical Emission Spectrometry (ICP-OES; iCap 7000 Series, Thermo Scientific). The measured nutrients (mg kg^-1^) included phosphorus (P), calcium (Ca), magnesium (Mg), potassium (K), iron (Fe), and manganese (Mn). Soil nitrogen (N) concentrations were determined by dry combustion.

*Soil Physical Properties*

Soil texture was determined on soil samples composited by sampling site using a (Gee and Bauder 2018) modified hydrometer method (Black 1965). Air-dried soil fractions < 2 mm were dispersed in 5% sodium hexametaphosphate (NaPO₃)₆ solution, shaken overnight, and then diluted to a 1 L suspension. A soil hydrometer (ASTM 152H) was inserted into the suspension, and hydrometer readings were taken at 40 seconds and 2 hours after thorough mixing, corresponding to the suspension densities of silt + clay and clay fractions, respectively. Temperature corrections were applied to all readings to account for deviations from the standard reference temperature (20°C). The percentages of sand, silt, and clay were calculated using the corrected hydrometer readings and sample weight. Soil textural classes were then assigned according to the USDA soil texture classification triangle.

*Soil Enzymatic Properties*

A high-throughput fluorometric assay method (German et al. 2011, Smith et al. 2015) was used to measure the potential extracellular enzyme activity of three key enzymes involved in nutrient cycling processes: β-glucosidase, N-acetylglucosaminidase (NAGase), and phosphatase. These enzymes are involved in the decomposition of compounds like cellulose and hemicellulose, chitin and N polymers, and in the acquisition of phosphorus, respectively. A soil slurry was prepared using 1 g freeze-dried soil in 100 ml tris(hydroxymethyl)aminomethane buffer (pH 7) and incubated at 23℃ for 1 h in the presence of fluorescent-linked (4-methylumbelliferone) 200 uM enzyme substrates in a 96-well plate. Reactions were stopped using 10 ul of 1M NaOH and plates were read on a Fluorescence-Microplate reader (Synergy, Biotek) at 360/40 nm and 460/40 nm excitation and emission wavelengths within 2 mins of stopping the reaction. Enzyme activities were normalized by SOC and calculated using the following equation (Smith et al. 2015):

$$Activity \left( \mathrm{mmol}{g SOC}^{-1} {hr}^{-1} \right)=\frac{Net Fluorescence \times Buffer Volue \times0.1}{Emission Coefficient \times Homogenate Volume \times Time \times Mass Soil Organic Carbon}$$

Net fluorescence was calculated as the difference in fluorescence among assay, slurry and buffer fluorescence and normalized by a quench coefficient. Outliers, data points 1.5 times the interquartile range, among technical replicates (i.e., 16 wells) were removed, and samples were rerun if the standard concentration curves for emission coefficients were not linear (i.e., R^2^ < 0.90) using R (version 1.2.1335).

*Library preparation and post-sequencing processing*

For each sample, we extracted total genomic DNA from approximately 250 mg of soil using the PowerSoil DNA Isolation Kit (Qiagen, USA). Following extraction, we assessed DNA quality using a NanoDrop Lite Spectrometer (Thermo Scientific, USA) and then followed a two-step process for library preparation (U’ren and Arnold 2017). Briefly, for the inner PCR, we amplified the regions of interest—the fungal internal transcribed spacer 1 (ITS1) region and the bacterial 16S V4-V5 region—and added Illumina adapters in triplicate. In the outer PCR, we added sample-specific barcodes. For fungal communities, we amplified the ITS1 region using primers ITS1F (Gardes and Bruns 1993) and ITS2 (White et al. 1990) amended with Illumina adapters (HybITS-1F_rRNA, 5'-TCGTCGGCAGCGTCAGATGTGTATAAGAGACAGCTTGGTCATTTAGAGGAAGTAA-3' and HybITS2_rRNA, 3'-CGTAGCTACTTCTTGCGTCGGACAGAGAATATGTGTAGAGGCTCGGGTGCTCTG-5'). For bacterial communities, we targeted the 16SrDNA V4-V5 region with primers 515F and 909R (Wang and Qian 2009) amended with Illumina adapters (Hyb515F_rRNA, 5- TCGTCGGCAGCGTCAGATGTGTATAAGAGACAGGTGYCAGCMGCCGCGGTA-3' and Hyb909R_rRNA, 3'-TGARTTTMCTTAACYGCCCCGACAGAGAATATGTGTAGAGGCTCGGGTGCTCTG-5'). After the inner PCR, we visualized PCR product on gels to confirm amplification and pooled the triplicate PCR product for each sample. We assessed DNA quality using NanoDrop Lite Spectrometer (Thermo Scientific, USA). Samples were then cleaned using AMPure Bead XP purification (Beckman Coulter, USA) based on standard protocols before quantifying the DNA concentrations on a Qubit 4 Fluorometer (Thermo Scientific, USA) using the Qubit dsDNA High Sensitivity Assay Kit. Sample DNA concentrations were normalized to 2 ng/ul for fungi and 3 ng/ul for bacteria. Samples were submitted to the University of Texas at Austin Genomic Sequencing and Analysis Facility (GSAF) for completion of the outer PCR and sequencing on Illumina MiSeq V2 PE250.

To limit the introduction and amplification of non-target DNA (i.e., environmental contamination), we decontaminated all surfaces and tools, including pipettes, with ethanol (95% EtOH) and DNase AWAY (Thermo Scientific, USA). For each step, we used aerosol-resistant pipette tips to precent cross sample contamination. We included extraction blanks and PCR blanks, which were carried through each step in parallel with the soil samples. We did not detect contamination when PCR product was imaged on gels, but we sequenced both sets of blanks along with samples. In addition to including blanks, we also included artificial communities to evaluate PCR and sequencing biases, to assess whether read abundance was correlated to expected biological abundance, and to identify OTU boundary thresholds and possible spurious OTUs (Nguyen et al. 2015). For the fungal artificial communities, we sequenced two artificial communities with each consisting of 32 species spanning major lineages of fungi. One community contained equal DNA concentrations of each species, and the second community had tiered or staggered concentrations (Bowman and Arnold 2021). For the bacterial artificial communities, we used a commercially available community, ZymoBIOMICS Microbial Community DNA Standard (Zymo Research, USA), which contains eight bacterial species and two fungal yeast species.

Following sequencing, reads were demultiplexed, and primers were removed from reads at GSAF using standard protocols. We processed raw reads with USEARCH v.10 (Edgar 2010). We merged paired reads (fungi: 2,883,233 contiguous sequences, 72.9%; bacteria: 2,384,338 contiguous sequences, 83.4%) and assessed read quality of merged reads with FastQC (Andrew 2010) and the -fastq_eestats2 command in USEARCH. We filtered and trimmed reads based on assessment of artificial communities and negative controls (fungi: maxEE 0.25, length 250 bp; bacteria: maxEE 0.5, length 250 bp) yielding 2,280,227 and 1,891,299 high quality reads for fungi and bacteria, respectively. We followed the processing steps outlined in Daru et al. (2019) and Bowman & Arnold (2021). Processing included isolating unique sequences (-fastx_uniques; fungi: 289,158; bacteria: 888,034); checking for and removing chimeras (-unoise3; fungi: 945; bacteria: 0) and potential sequencing errors (fungi and bacteria: sequences with < 50 occurrences); and clustering OTUs based on sequence similarity determine by field standards and assessment of the artificial communities (-cluster_smallmem; fungi: 95%; bacteria: 99%). Additionally, we clustered both datasets at 90% sequence similarity for combined analyses (e.g., network and co-occurrence analyses; see below).

All major expected lineages were amplified in the artificial communities, indicating no primer bias. The two yeast fungi in the ZymoBIOMICS Microbial Community DNA Standard were not amplified in the bacterial dataset. Based on assessment of the artificial communities, we rarefied the fungal reads with coverage-based rarefaction to minimize uneven coverage of samples without underestimating richness (Chao and Jost 2012, Okazaki et al. 2017). When we rarefied fungal reads and filtered bacterial reads as above, read abundance matched the expected abundance of each species in the artificial communities we used, indicating that read abundance is an acceptable proxy for biological abundance in our dataset.

Taxonomic placement of fungal sequence data was estimated by querying GenBank (Altschul et al. 1990) with validation in UNITE (Kõljalg et al. 2013, Nilsson et al. 2019). Taxonomy of bacterial sequences was assigned using the q2-feature-classifier in QIIME 2 (Bolyen et al. 2019) and SILVA 16S rRNA gene database (Quast et al. 2013). The classifier was trained using a naïve Bayes machine-learning classifier for non-saline soil (<https://zenodo.org/record/6395539>) (Bokulich et al. 2018, Kaehler et al. 2019, Robeson et al. 2021).

**References**

Altschul SF, Gish W, Miller W, Myers EW, Lipman DJ. 1990. Basic Local Alignment Search Tool 2Department of Computer Science. *Journal of Molecular Biology* 215: 403–410.

Andrew S. 2010. FastQC: a quality control tool for high throughput sequence data.

Black CA (Ed.). 1965. Methods of Soil Analysis. 9.

Bokulich NA, Kaehler BD, Rideout JR, Dillon MR, Bolyen E, Knight R, Huttley GA, Caporaso JG. 2018. Optimizing taxonomic classification of marker-gene amplicon sequences with QIIME 2’s q2-feature-classifier plugin. *Microbiome* 6.

Bolyen E, Rideout JR, Dillon MR, Bokulich NA, Abnet CC, Al-Ghalith GA, Alexander H, Alm EJ, Arumugam M, Asnicar F, *et al.* 2019. Reproducible, interactive, scalable and extensible microbiome data science using QIIME 2. *Nature Biotechnology* 37: 852–857.

Bowman EA, Arnold AE. 2021. Drivers and implications of distance decay differ for ectomycorrhizal and foliar endophytic fungi across an anciently fragmented landscape. *The ISME Journal*: 8–12.

Chao A, Jost L. 2012. Coverage-based rarefaction and extrapolation: standardizing samples by completeness rather than size. *Ecology* 93: 2533–2547.

Culman SW, Snapp SS, Freeman MA, Schipanski ME, Beniston J, Lal R, Drinkwater LE, Franzluebbers AJ, Glover JD, Grandy AS, *et al.* 2012. Permanganate Oxidizable Carbon Reflects a Processed Soil Fraction that is Sensitive to Management. *Soil Science Society of America Journal* 76: 494–504.

Daru BHBH, Bowman EAEA, Pfister DHDH, Arnold AEE. 2019. A novel proof of concept for capturing the diversity of endophytic fungi preserved in herbarium specimens. *Philosophical Transactions of the Royal Society B: Biological Sciences* 374.

Dhillon GS, Amichev BY, de Freitas R, Van Rees K. 2015. Accurate and Precise Measurement of Organic Carbon Content in Carbonate-Rich Soils. *Communications in Soil Science and Plant Analysis* 46: 2707–2720.

Edgar RC. 2010. Search and clustering orders of magnitude faster than BLAST. *Bioinformatics* 26: 2460–2461.

Gardes M;, Bruns TD. 1993. ITS primers with enhanced specificity for basidiomycetes - application to the identificiation of mycorrhizae and rusts. *Molecular Ecology* 2: 113–118.

Gee GW, Bauder JW. 2018. *Particle-size Analysis*. John Wiley & Sons, Ltd.

German DP, Weintraub MN, Grandy AS, Lauber CL, Rinkes ZL, Allison SD. 2011. Optimization of hydrolytic and oxidative enzyme methods for ecosystem studies. *Soil Biology and Biochemistry* 43: 1387–1397.

Harris D, Horwáth WR, van Kessel C. 2001. Acid fumigation of soils to remove carbonates prior to total organic carbon or CARBON‐13 isotopic analysis. *Soil Science Society of America Journal* 65: 1853–1856.

Kaehler BD, Bokulich NA, McDonald D, Knight R, Caporaso JG, Huttley GA. 2019. Species abundance information improves sequence taxonomy classification accuracy. *Nature Communications* 10: 1–10.

Kõljalg U, Nilsson RH, Abarenkov K, Tedersoo L, Taylor AFS, Bahram M, Bates ST, Bruns TD, Bengtsson-Palme J, Callaghan TM, *et al.* 2013. Towards a unified paradigm for sequence-based identification of fungi. *Molecular Ecology* 22: 5271–5277.

Mehlich A. 1984. Mehlich 3 Soil Test Extractant: A Modification of Mehlich 2 Extractant. *Communications in Soil Science and Plant Analysis* 15: 1409–1416.

Mishra NB, Young KR. 2020. Savannas and Grasslands. In: Wang Y, ed. Terrestrial Ecosystems and Biodiversity. Boca Raton: CRC Press, 235–247.

Nguyen NH, Smith D, Peay K, Kennedy P. 2015. Parsing ecological signal from noise in next generation amplicon sequencing. *New Phytologist* 205: 1389–1393.

Nilsson RH, Larsson KH, Taylor AFS, Bengtsson-Palme J, Jeppesen TS, Schigel D, Kennedy P, Picard K, Glöckner FO, Tedersoo L, *et al.* 2019. The UNITE database for molecular identification of fungi: Handling dark taxa and parallel taxonomic classifications. *Nucleic Acids Research* 47: D259–D264.

Okazaki Y, Fujinaga S, Tanaka A, Kohzu A, Oyagi H, Nakano SI. 2017. Ubiquity and quantitative significance of bacterioplankton lineages inhabiting the oxygenated hypolimnion of deep freshwater lakes. *ISME Journal* 11: 2279–2293.

Quast C, Pruesse E, Yilmaz P, Gerken J, Schweer T, Yarza P, Peplies J, Glöckner FO. 2013. The SILVA ribosomal RNA gene database project: Improved data processing and web-based tools. *Nucleic Acids Research* 41: 590–596.

Robeson MS, O’Rourke DR, Kaehler BD, Ziemski M, Dillon MR, Foster JT, Bokulich NA. 2021. *RESCRIPt: Reproducible sequence taxonomy reference database management*.

Smith AP, Marín-Spiotta E, Balser T. 2015. Successional and seasonal variations in soil and litter microbial community structure and function during tropical postagricultural forest regeneration: A multiyear study. *Global Change Biology* 21: 3532–3547.

Soil Survey Staff, Natural Resources Conservation Service, United States Department of Agriculture. 2024. Official Soil Series Descriptions. *Available Online*.

Texas Geographic Information Office tnris. org. 2022. Historical Imagery Archive. *Texas Geographic Information System (https://tnris.org/research-distribution-center/historical-imagery-archive.html)*.

U’ren JM, Arnold AE. 2017. Illumina MiSeq Dual-barcoded Two-step PCR Amplicon Sequencing Protocol. : 1–7.

Wang Y, Qian PY. 2009. Conservative fragments in bacterial 16S rRNA genes and primer design for 16S ribosomal DNA amplicons in metagenomic studies. *PLoS ONE* 4.

Weil RR, Islam KR, Stine MA, Gruver JB, Samson-Liebig SE. 2003. Estimating active carbon for soil quality assessment: A simplified method for laboratory and field use. *American Journal of Alternative Agriculture* 18: 3–17.

White T, Bruns TD, Lee S, Taylor J. 1990. Amplification and direct sequencing of fungal ribosomal RNA genes for phylogenetics. In: Innis N, Gelfand D, Sninsky J, White T, eds. PCR Protocols: A Guide to Methods and Applications. Academic Press, 315–322.
