# Supplementary material for: Soil Microbial Assembly Mirrors a Savanna Mosaic Amid Plant Invasion and Soil Disturbance
Source: Ecol Evol. 2026 Sep 3;16(9):e74284. doi: 10.1002/ece3.74284 (PMC13540272; doi:10.1002/ece3.74284)
Supplement: Supplementary file 2 [file ECE3-16-e74284-s002.docx]

**Appendix S2: Supplemental Figures and Tables**

**Supplementary Figure S1:** Mean annual precipitation (MAP) varied 6.7 mm across sampling sites.

**Supplementary Figure S2:** Histogram of percent cover of *M. maximus* across all sites (a) and percent cover as a function of vegetation type in invaded sites (b). In panel a, each sample is colored by its invasion status (white = uninvaded; blue-green = invaded).

**Supplementary Figure S3:** Plant species richness and diversity were higher in grasslands (a) and uninvaded sites (b). Here, only plant species richness is shown.

**Supplementary Figure S4:** PCA of soil traits as a function of invasion (a) and disturbance (b). Boxplot of PC1 as a function of vegetation type (c), invasion (d), and disturbance (e). The point colors match those in the corresponding boxplots. Panel a colors match those in panel d; panel b colors match those in panel e.

**Supplementary Figure S5:** Invasion and disturbance had an interactive effect on bacterial OTU richness (a). Bacterial diversity was higher in grasslands (b) NMDS of fungal (c; stress = 0.23) and bacterial (d; stress = 0.12) communities across all sites. The dissimilarity indices represented here are Morisita-Horn and Jaccard, respectively.

**Supplementary Figure S6:** NMDS of fungal communities (a, b) and bacterial communities (c, d) in grasslands (a, c) and woody patches (b, d). The dissimilarity index used in panels a (stress = 0.23) and b (stress = 0.26) is the Morisita-Horn and the Jaccard in panels c (stress = 0.12) and d (stress = 0.11).

**Supplementary Table S1:** List of sampling sites and associated metadata. Climate data was downloaded from WorldClim (Fick & Hijmans, 2017). Disturbance year is the most recent brush management event.

| Site | Disturbance | Invasion | Vegetation | Latitude | Longitude | MAT (°C) | MAP (mm) |
| --- | --- | --- | --- | --- | --- | --- | --- |
| 1 | 2004 | Invaded | Grassland matrix | 27.22223 | -97.97246 | 22.3 | 649.4 |
| 1 | 2004 | Invaded | Woody patch | 27.22276 | -97.97208 | 22.3 | 649.5 |
| 1 | 2004 | Uninvaded | Grassland matrix | 27.22256 | -97.97251 | 22.3 | 649.5 |
| 1 | 2004 | Uninvaded | Woody patch | 27.23439 | -97.98122 | 22.3 | 651.1 |
| 1 | >1980 | Invaded | Grassland matrix | 27.22518 | -97.96783 | 22.3 | 649.7 |
| 1 | >1980 | Invaded | Woody patch | 27.2219 | -97.966 | 22.3 | 649.1 |
| 1 | >1980 | Uninvaded | Grassland matrix | 27.22492 | -97.96741 | 22.3 | 649.6 |
| 1 | >1980 | Uninvaded | Woody patch | 27.22202 | -97.966 | 22.3 | 649.1 |
| 2 | 1998 | Invaded | Grassland matrix | 27.18099 | -97.96601 | 22.2 | 648.4 |
| 2 | 1998 | Invaded | Woody patch | 27.18693 | -97.96643 | 22.3 | 649.0 |
| 2 | 1998 | Uninvaded | Grassland matrix | 27.1862 | -97.96653 | 22.3 | 648.9 |
| 2 | 1998 | Uninvaded | Woody patch | 27.18621 | -97.96992 | 22.2 | 649.1 |
| 2 | >1943 | Invaded | Grassland matrix | 27.18643 | -97.9663 | 22.3 | 648.9 |
| 2 | >1943 | Invaded | Woody patch | 27.18114 | -97.96597 | 22.2 | 648.4 |
| 2 | >1943 | Uninvaded | Grassland matrix | 27.18087 | -97.96629 | 22.2 | 648.5 |
| 2 | >1943 | Uninvaded | Woody patch | 27.18115 | -97.96615 | 22.2 | 648.5 |
| 3 | 1995 | Invaded | Grassland matrix | 27.20874 | -97.88339 | 22.3 | 655.1 |
| 3 | 1995 | Invaded | Woody patch | 27.20873 | -97.8835 | 22.3 | 655.1 |
| 3 | 1995 | Uninvaded | Grassland matrix | 27.20908 | -97.88348 | 22.3 | 655.2 |
| 3 | 1998 | Uninvaded | Woody patch | 27.19058 | -97.96037 | 22.3 | 648.6 |
| 3 | >1980 | Invaded | Grassland matrix | 27.20061 | -97.88795 | 22.3 | 652.7 |
| 3 | >1980 | Invaded | Woody patch | 27.202522 | -97.886558 | 22.3 | 653.4 |
| 3 | >1980 | Uninvaded | Grassland matrix | 27.2007 | -97.88773 | 22.3 | 652.7 |
| 3 | >1980 | Uninvaded | Woody patch | 27.20273 | -97.88614 | 22.3 | 653.5 |

**Table S2:**

| **Response variable** | **Fixed effects** | **Random effect** | **Sample size** | **Notes** |
| --- | --- | --- | --- | --- |
| *Overall analyses* | | | | |
| Guinea grass cover in invaded areas | Plant community * disturbance | block/plot | 24 |  |
| Plant species richness and diversity | Plant community * disturbance * invasion | block/plot | 48 |  |
| Soil characteristics |  | block/plot | 120 |  |
| Fungal species richness and diversity |  | block/plot | 120 |  |
| Fungal community composition |  | block | 120 | Removed OTU with < 1 read (singletons) |
| Bacterial species richness and diversity |  | block/plot | 120 |  |
| Bacterial community composition |  | block | 120 | Removed OTU with < 10 reads |
| Taxonomic analyses | Plant community |  | 120 |  |
| *Grassland analyses* | | | | |
| Soil enzymes | Fungal diversity + bacterial diversity + plant Shannon's residuals + PC1.soil | block/plot | 59 | Phosphatase was square root transformed. |
| Fungal OTU richness and diversity | Plant Shannon's residuals + PC1.soil | block/plot | 59 |  |
| Fungal community composition | PC1.soil * plant Shannon's residuals + PCNM1 | block | 59 | Removed OTU with < 1 read (singletons) |
| Bacterial OTU richness and diversity | plant Shannon's residuals + PC1.soil | block/plot | 59 |  |
| Bacterial community composition | PC1.soil * plant Shannon's residuals + PCNM1 | block | 59 | Removed OTU with < 10 reads |
| *Woody patch analyses* | | | | |
| Soil enzymes | Fungal diversity + bacterial diversity + plant Shannon's diversity + PC1.soil | block/plot | 60 |  |
| Fungal OTU richness and diversity | Plant Shannon's diversity + PC2.soil | block/plot | 60 |  |
| Fungal community composition | PC1.soil * plant Shannon's residuals + PCNM1 | block | 60 | Removed OTU with < 1 read (singletons) |
| Bacterial OTU richness and diversity | Plant Shannon's diversity + PC2.soil | block/plot | 60 | Bacterial OTU richness was square root transformed |
| Bacterial community composition | PC1.soil * plant Shannon's residuals + PCNM1 | block | 60 | Removed OTU with < 10 reads |

**Supplementary Table S3:** Mean and standard error of soil enzymatic and chemical properties (A). Mean and standard error of soil nutrients (B).

**A.**

| **Vegetation** | **Plot** | **Disturbance** | **Invasion** | **Beta**  umol gSOC^-1^h^-1^ | **Nag**  umol gSOC^-1^h^-1^ | **Phos**  umol gSOC^-1^h^-1^ | **SOC** % | **Active C** mg kg^-1^ | **N** % | **C:N** | **pH** |
| --- | --- | --- | --- | --- | --- | --- | --- | --- | --- | --- | --- |
| Grassland | 1 | Disturbed | Invaded | 18997 ± 4309 | 5785 ± 3581 | 35687 ± 27959 | 0.59 ± 0.1 | 993 ± 55 | 0.064 ± 0.01 | 9.2 ± 0.7 | 7.0 ± 1.1 |
|  |  |  | Uninvaded | 10757 ± 2295 | 4642 ± 1356 | 55165 ± 29985 | 0.49 ± 0.1 | 980 ± 40 | 0.054 ± 0.01 | 9.0 ± 0.7 | 6.0 ± 0.8 |
|  |  | Undisturbed | Invaded | 15262 ± 6789 | 4860 ± 2922 | 54168 ± 28066 | 0.59 ± 0.2 | 995 ± 47 | 0.062 ± 0.02 | 9.6 ± 0.7 | 6.1 ± 0.4 |
|  |  |  | Uninvaded | 10451 ± 11152 | 2676 ± 2912 | 47744 ± 49028 | 0.47 ± 0.0 | 945 ± 45 | 0.057 ± 0.01 | 8.5 ± 1.6 | 5.5 ± 0.7 |
|  | 2 | Disturbed | Invaded | 14374 ± 6228 | 2285 ± 1435 | 22711 ± 14629 | 0.79 ± 0.2 | 1057 ± 27 | 0.081 ± 0.02 | 9.6 ± 0.9 | 7.0 ± 1.1 |
|  |  |  | Uninvaded | 14481 ± 10636 | 4592 ± 4075 | 35155 ± 23383 | 0.52 ± 0.1 | 1052 ± 103 | 0.053 ± 0.01 | 9.8 ± 1.1 | 6.1 ± 0.3 |
|  |  | Undisturbed | Invaded | 19507 ± 10216 | 6268 ± 5169 | 61227 ± 23626 | 0.47 ± 0.1 | 987 ± 53 | 0.052 ± 0.01 | 9.2 ± 0.7 | 5.6 ± 0.5 |
|  |  |  | Uninvaded | 12899 ± 5687 | 5140 ± 3476 | 42802 ± 44097 | 0.41 ± 0.0 | 974 ± 52 | 0.057 ± 0.01 | 7.4 ± 1.3 | 5.4 ± 0.4 |
|  | 3 | Disturbed | Invaded | 21012 ± 11766 | 5789 ± 2941 | 49244 ± 25646 | 0.68 ± 0.2 | 1009 ± 40 | 0.069 ± 0.02 | 9.8 ± 0.5 | 6.5 ± 0.5 |
|  |  |  | Uninvaded | 19120 ± 5354 | 4461 ± 2345 | 34946 ± 17112 | 0.50 ± 0.1 | 1016 ± 18 | 0.052 ± 0.01 | 9.6 ± 0.9 | 6.4 ± 0.4 |
|  |  | Undisturbed | Invaded | 14973 ± 3867 | 4310 ± 1784 | 55311 ± 13684 | 0.53 ± 0.1 | 994 ± 92 | 0.055 ± 0.01 | 9.6 ± 0.8 | 6.4 ± 0.4 |
|  |  |  | Uninvaded | 20460 ± 11896 | 6070 ± 4058 | 58662 ± 26128 | 0.44 ± 0.1 | 984 ± 47 | 0.054 ± 0.02 | 8.6 ± 1.6 | 6.3 ± 0.7 |
| Woody patch | 1 | Disturbed | Invaded | 10629 ± 3697 | 2364 ± 1080 | 18035 ± 10012 | 1.65 ± 0.7 | 1040 ± 63 | 0.170 ± 0.08 | 9.8 ± 0.4 | 6.7 ± 0.7 |
|  |  |  | Uninvaded | 17482 ± 11993 | 4060 ± 1151 | 32869 ± 29411 | 0.96 ± 0.2 | 1039 ± 27 | 0.112 ± 0.02 | 8.6 ± 0.8 | 7.2 ± 0.3 |
|  |  | Undisturbed | Invaded | 12783 ± 6886 | 5305 ± 3549 | 54305 ± 33543 | 0.61 ± 0.1 | 1018 ± 43 | 0.064 ± 0.01 | 9.5 ± 0.2 | 5.5 ± 0.3 |
|  |  |  | Uninvaded | 7273 ± 2206 | 2358 ± 679 | 27041 ± 10763 | 0.88 ± 0.3 | 1022 ± 58 | 0.097 ± 0.03 | 9.1 ± 0.7 | 6.4 ± 0.8 |
|  | 2 | Disturbed | Invaded | 8837 ± 3224 | 2394 ± 1063 | 10484 ± 5785 | 1.49 ± 0.4 | 1078 ± 46 | 0.157 ± 0.03 | 9.4 ± 0.4 | 6.6 ± 0.7 |
|  |  |  | Uninvaded | 5874 ± 2881 | 1618 ± 890 | 16879 ± 16138 | 0.92 ± 0.3 | 982 ± 54 | 0.101 ± 0.03 | 9.1 ± 0.3 | 6.6 ± 1.2 |
|  |  | Undisturbed | Invaded | 3744 ± 3613 | 1579 ± 1312 | 9218 ± 6830 | 1.25 ± 0.5 | 1005 ± 65 | 0.116 ± 0.04 | 10.7 ± 0.8 | 5.9 ± 0.5 |
|  |  |  | Uninvaded | 8150 ± 10074 | 3333 ± 2744 | 15289 ± 18919 | 0.70 ± 0.0 | 1032 ± 80 | 0.068 ± 0.01 | 10.2 ± 0.5 | 5.0 ± 0.4 |
|  | 3 | Disturbed | Invaded | 9114 ± 5061 | 5513 ± 2161 | 32912 ± 27526 | 0.82 ± 0.2 | 1033 ± 52 | 0.083 ± 0.02 | 9.9 ± 0.3 | 5.3 ± 0.3 |
|  |  |  | Uninvaded | 12800 ± 7368 | 2411 ± 1314 | 20693 ± 17263 | 1.01 ± 0.3 | 1042 ± 21 | 0.112 ± 0.04 | 9.1 ± 0.5 | 6.5 ± 0.5 |
|  |  | Undisturbed | Invaded | 8599 ± 3931 | 3853 ± 1594 | 24378 ± 12731 | 0.95 ± 0.2 | 1040 ± 54 | 0.102 ± 0.02 | 9.4 ± 0.4 | 5.8 ± 0.6 |
|  |  |  | Uninvaded | 12885 ± 6260 | 2999 ± 680 | 20264 ± 11565 | 1.12 ± 0.4 | 1030 ± 63 | 0.126 ± 0.04 | 8.8 ± 0.8 | 6.1 ± 1.1 |

**B.**

| **Vegetation** | **Plot** | **Disturbance** | **Invasion** | **C**  (mg kg^-1^) | **N**  (mg kg^-1^) | **P**  (mg kg^-1^) | **Mg**  (mg kg^-1^) | **Mn**  (mg kg^-1^) | **Fe**  (mg kg^-1^) | **K**  (mg kg^-1^) | **Ca**  (mg kg^-1^) |
| --- | --- | --- | --- | --- | --- | --- | --- | --- | --- | --- | --- |
| Grassland | 1 | Disturbed | Invaded | 5920 ± 672 | 642 ± 63 | 223 ± 64 | 1513 ± 650 | 518 ± 245 | 575 ± 279 | 2045 ± 513 | 9091 ± 2840 |
|  |  |  | Uninvaded | 4896 ± 906 | 544 ± 80 | 152 ± 10 | 960 ± 330 | 502 ± 185 | 830 ± 323 | 1818 ± 554 | 4558 ± 1508 |
|  |  | Undisturbed | Invaded | 5946 ± 2015 | 618 ± 208 | 162 ± 34 | 874 ± 213 | 375 ± 41 | 672 ± 68 | 1508 ± 254 | 4412 ± 1500 |
|  |  |  | Uninvaded | 4722 ± 429 | 570 ± 89 | 102 ± 11 | 779 ± 67 | 336 ± 36 | 673 ± 81 | 1353 ± 93 | 4393 ± 802 |
|  | 2 | Disturbed | Invaded | 7862 ± 2472 | 810 ± 199 | 239 ± 123 | 1699 ± 390 | 450 ± 38 | 567 ± 158 | 2315 ± 417 | 10365 ± 3065 |
|  |  |  | Uninvaded | 5223 ± 1097 | 533 ± 96 | 116 ± 20 | 857 ± 165 | 307 ± 40 | 630 ± 111 | 1472 ± 261 | 4976 ± 991 |
|  |  | Undisturbed | Invaded | 4710 ± 927 | 520 ± 127 | 198 ± 94 | 976 ± 433 | 425 ± 154 | 782 ± 45 | 1447 ± 461 | 5511 ± 3149 |
|  |  |  | Uninvaded | 4080 ± 378 | 569 ± 139 | 173 ± 8 | 519 ± 75 | 204 ± 54 | 1188 ± 112 | 802 ± 136 | 2544 ± 498 |
|  | 3 | Disturbed | Invaded | 6812 ± 1870 | 691 ± 175 | 157 ± 34 | 1419 ± 586 | 658 ± 309 | 582 ± 235 | 2091 ± 1077 | 7510 ± 2631 |
|  |  |  | Uninvaded | 4964 ± 1184 | 516 ± 104 | 167 ± 31 | 1001 ± 314 | 241 ± 126 | 861 ± 398 | 1561 ± 635 | 6631 ± 2052 |
|  |  | Undisturbed | Invaded | 5282 ± 645 | 552 ± 57 | 146 ± 22 | 902 ± 155 | 505 ± 46 | 499 ± 64 | 1544 ± 213 | 5708 ± 1269 |
|  |  |  | Uninvaded | 4402 ± 614 | 544 ± 210 | 109 ± 7 | 797 ± 131 | 371 ± 54 | 484 ± 84 | 1392 ± 169 | 4515 ± 808 |
| Woody patch | 1 | Disturbed | Invaded | 16543 ± 7285 | 1695 ± 768 | 513 ± 95 | 3082 ± 1277 | 601 ± 114 | 477 ± 93 | 4330 ± 534 | 17773 ± 8852 |
|  |  |  | Uninvaded | 9574 ± 1862 | 1121 ± 224 | 170 ± 51 | 1642 ± 259 | 599 ± 69 | 318 ± 54 | 2634 ± 635 | 13353 ± 3316 |
|  |  | Undisturbed | Invaded | 6107 ± 866 | 640 ± 79 | 200 ± 116 | 842 ± 243 | 369 ± 46 | 657 ± 63 | 1265 ± 228 | 6472 ± 1598 |
|  |  |  | Uninvaded | 8770 ± 2698 | 966 ± 286 | 156 ± 25 | 1046 ± 80 | 349 ± 63 | 588 ± 55 | 1620 ± 216 | 10240 ± 3300 |
|  | 2 | Disturbed | Invaded | 14850 ± 3796 | 1572 ± 339 | 370 ± 138 | 2792 ± 977 | 704 ± 58 | 405 ± 159 | 3311 ± 663 | 19799 ± 7178 |
|  |  |  | Uninvaded | 9162 ± 3134 | 1012 ± 351 | 288 ± 111 | 2214 ± 1626 | 541 ± 61 | 568 ± 334 | 2197 ± 812 | 17957 ± 14730 |
|  |  | Undisturbed | Invaded | 12534 ± 5080 | 1160 ± 397 | 293 ± 127 | 1334 ± 500 | 472 ± 55 | 772 ± 146 | 1737 ± 247 | 8527 ± 3344 |
|  |  |  | Uninvaded | 6978 ± 239 | 682 ± 36 | 307 ± 64 | 572 ± 100 | 123 ± 68 | 1348 ± 192 | 867 ± 146 | 3517 ± 911 |
|  | 3 | Disturbed | Invaded | 8162 ± 1692 | 831 ± 192 | 330 ± 70 | 1315 ± 519 | 348 ± 79 | 560 ± 38 | 1899 ± 737 | 7906 ± 3233 |
|  |  |  | Uninvaded | 10138 ± 3372 | 1116 ± 382 | 394 ± 168 | 1946 ± 1003 | 500 ± 102 | 539 ± 138 | 2108 ± 481 | 14607 ± 10252 |
|  |  | Undisturbed | Invaded | 9464 ± 1997 | 1016 ± 236 | 270 ± 144 | 1190 ± 900 | 390 ± 263 | 397 ± 237 | 1529 ± 837 | 11357 ± 8915 |
|  |  |  | Uninvaded | 11187 ± 4081 | 1259 ± 384 | 295 ± 130 | 2128 ± 1151 | 503 ± 101 | 488 ± 187 | 2299 ± 502 | 16451 ± 9471 |

**Supplementary Table S4:** Plant species richness and diversity were higher in uninvaded sites and grasslands.

|  | Species richness | | Shannon's diversity | |
| --- | --- | --- | --- | --- |
| Variable | F-value | *p* | F-value | *p* |
| Invasion | 45.9 | **< 0.0001** | 80.8 | **< 0.0001** |
| Disturbance | 0.2 | 0.455 | 0.8 | 0.3796 |
| Vegetation type | 22.9 | **0.0003** | 13.2 | **0.0022** |
| Inv. * Dist. | 3.2 | 0.0930 | 2.2 | 0.1585 |
| Inv. * Veg. | 3.7 | 0.0746 | 0.0 | 0.9509 |
| Dist. * Veg. | 0.2 | 0.6701 | 0.0 | 0.8571 |
| Inv. * Dist. * Inv. | 0.9 | 0.3670 | 0.1 | 0.7917 |
